# Global nutritional equity of fishmeal and aquaculture trade flows

**DOI:** 10.1073/pnas.2506699123

**Published:** 2026-02-09

**Authors:** Laura G. Elsler, Jessica A. Gephart, Jessica Zamborain-Mason, Tim Cashion, Max Troell, Rosamond L. Naylor, Rahul Agrawal Bejarano, Christopher D. Golden

**Affiliations:** ^a^Department of Nutrition, Harvard T.H. Chan School of Public Health, Boston, MA 02115; ^b^Department of Environmental Health, Harvard T.H. Chan School of Public Health, Boston, MA 02115; ^c^School of Aquatic and Fishery Sciences, University of Washington, Seattle, WA 98195; ^d^Biological and Environmental Science and Engineering Division, King Abdullah University of Science and Technology, Thuwal 23955, Saudi Arabia; ^e^Lancaster Environment Centre, Lancaster University, Lancaster LA1 4YQ, United Kingdom; ^f^Institute for Resources, Environment and Sustainability, The University of British Columbia, Vancouver, BC V6T 1Z4, Canada Resilience Centre, Stockholm University, Stockholm 11418, Sweden; ^g^Fisheries Economics Research Unit, Institute for the Oceans and Fisheries, The University of British Columbia, Vancouver, BC V6T 1Z4, Canada; ^h^The Beijer Institute and Global Economic Dynamics and the Biosphere (GEDB), Royal Swedish Academy of Sciences, Stockholm 11418, Sweden; ^i^Stockholm Resilience Centre, Stockholm University, Stockholm 11418, Sweden; ^j^Department of Environmental Social Sciences, Stanford Doerr School of Sustainability, Stanford University, Stanford, CA 94305

**Keywords:** seafood markets, globalization, environmental justice, aquatic food systems, inequality

## Abstract

Aquaculture, the single fastest growing food sector, is central to achieving key Sustainable Development Goals (e.g., SDG 2: Zero Hunger). Linking the nutrient composition of >2,800 aquatic species with >2 million fishmeal and farmed fish transactions in international aquatic food trade between 2015 and 2019, we examined aquaculture’s nutritional flows and distributional equity. We found that aquaculture provided adequate intakes for nearly a quarter of a million individuals, on average, across 14 key nutrients, and for up to 2.7 billion individuals for several nutrients, such as Vitamin B_12_. The vast majority of these nutrients (76.8%) were domestically retained, contributing to the nutritional security of producer countries. With most internationally traded nutrients originating from nutritionally vulnerable countries (57.7% for fishmeal and 66.3% for farmed aquatic foods), rethinking existing distribution policies with nutrition as the primary objective may help unlock the full potential of aquaculture to eliminate hunger and malnutrition.

## Significance

Aquaculture’s rapid growth and trade development, combined with its continued reliance on capture fisheries, have yet unknown consequences for how, where, and under which conditions aquaculture can equitably enhance human health and nutrition. We found that high domestic retention of farmed aquatic foods is a substantial contribution to domestic nutrition. Yet, the traded portion of fishmeal and farmed aquatic foods is primarily sourced from nutritionally vulnerable areas. These findings highlight that nutrition-sensitive trade and development policies have the potential to enhance aquaculture’s contribution to attaining Sustainable Development Goal 2.

Aquatic foods provide critical nutrients to billions of people globally and are expected to be central in the pursuit of the Sustainable Development Goals (SDGs), in particular Goal 2, Zero Hunger (1–3). Aquatic food consumption has doubled in the past 50 y, exceeding 20.7 kg live weight per capita in 2022 (4). Global aquaculture production increased more than threefold in aquatic animal live weight from 1997 to 2017 (5), rivaling capture fisheries as a primary source of aquatic food for direct human consumption (4).

Aquaculture can contribute to nutritional security directly and indirectly (3). Farmed aquatic foods can directly improve food availability and access for consumers if they drive prices down (6) and are available to a large share of the population (7). Policies to increase aquaculture production often aim to compensate for declining fishery catch and promote food and nutritional security (8). For example, in Bangladesh, increased production alongside retaining fish for domestic consumption rather than exporting contributes to increased aquatic food availability (9). In addition, economic benefits can indirectly lead to positive nutritional benefits for small-scale aquaculture producers by generating higher incomes (10) and by providing access to high-value markets (6), such as the lobster and abalone cooperatives of Baja California (11).

Increasing aquaculture production alone does not guarantee improved food and nutritional outcomes (12, 13). Food security rests on pillars of sustainability and access in addition to increased production (14). Despite efficiency gains for many species and the increasing use of by-products and trimmings (4, 5), the aquaculture sector continues to rely on production from land and sea (15). Feed composition has increasingly shifted toward terrestrial crop inputs (16, 17), yet overall reliance on nutritionally rich, wild-caught fish and invertebrates also remains high (18, 19), threatening the sector’s sustainability. Second, international trade mediates access to aquatic foods. Recent research has shown that trade potentially exacerbates nutritional inequities in aquatic food consumption (20, 21). For instance, least developed countries (as defined by the United Nations) tend to be net aquatic food exporters, with exports nearly tripling in the past two decades (22). This means while food trade and production can support equitable nutrient distribution (23), it remains unclear whether this is true across the diversity of farmed aquatic species or for aquaculture as a whole.

To measure nutrient production and distribution across aquaculture supply chains, our study merged two novel global databases: the Aquatic Resource Trade in Species [ARTIS; (24)] and the Aquatic Food Composition Database [AFCD; (25)]. Using average values for 2015 to 2019, we examined i) the nutrient supply and equity of nutrient distribution of global aquaculture production and trade flows from and to nutritionally vulnerable countries (where trade accounts for international transactions of fishmeal and farmed fish and invertebrates, henceforth farmed fish), and ii) the potential nutritional benefits from hypothetically repurposing for direct human consumption and domestically retaining exports, addressing the question of which countries may nutritionally benefit from retaining exports for their own consumption. We measured country nutritional vulnerability by the prevalence of inadequate nutrient intake (26). We converted edible weight (defined as species muscle tissue) to annual recommended dietary allowances (RDAs) of women of reproductive age, a group of particular importance in public health, to calculate the total supply of individual nutrient needs produced (Methods 4.2). We included 14 nutrients important for human health: calcium, folate, iodine, iron, magnesium, niacin, selenium, thiamin, vitamin A, vitamin B_2_, vitamin B_6_, vitamin B_12_, vitamin E, and zinc (27). Finally, we highlight nutritionally vulnerable regions that would benefit the most from a shift in fishmeal and aquaculture trade flows.

## 1. Results and Discussion

### 1.1. Global Aquaculture Nutrient Production.

On average across the studied nutrients and between 2015 and 2019, the global aquaculture sector produced aquatic food equivalent to the annual nutrient needs of 347 million individuals (*SI Appendix*, Table S1). These values assume a diet consisting only of aquatic foods, whereas they are part of a bigger food portfolio. Minimum diversity indicators for tracking SDGs are a proxy for micronutrient adequacy in the diet, with consumption of at least five food groups for women of reproductive age being adequate (28). Thus, if aquatic foods are considered as one of the five food groups consumed, the number of people for whom annual nutrient needs would potentially be met would increase fivefold. Considering specific nutrients, global aquaculture production can meet the individual nutrient needs of 2.7 billion people for vitamin B12, niacin (521 million people), and selenium (467 million people) (*SI Appendix*, Table S2). In comparison, inadequate intake of vitamin B_12_, niacin, and selenium affected 3.0, 1.7, and 2.8 billion individuals.

Aquaculture produced on average 36.1 million tons of edible weight annually (corresponding to 79 million tons of live weight), accounting for 42.5% of total aquatic food (i.e., aquaculture and capture fisheries combined). Measured in individual nutrient needs (average across the studied nutrients), aquaculture accounted for 41.1% of total aquatic food, a lower proportion than edible weight. However, some nutrients contributed more in terms of nutrient supply than edible weight, including thiamin (52.5%), folate (51.9%), vitamin E (49.8%), vitamin B_12_ (44.7%), calcium (43.6%), and zinc (43.4%) The percentage difference between edible weight and nutrients is driven by variability in aquatic species’ nutrient profiles and the species composition of production, demonstrating the nutritional superiority of capture fisheries on average.

**Fig. 1.**
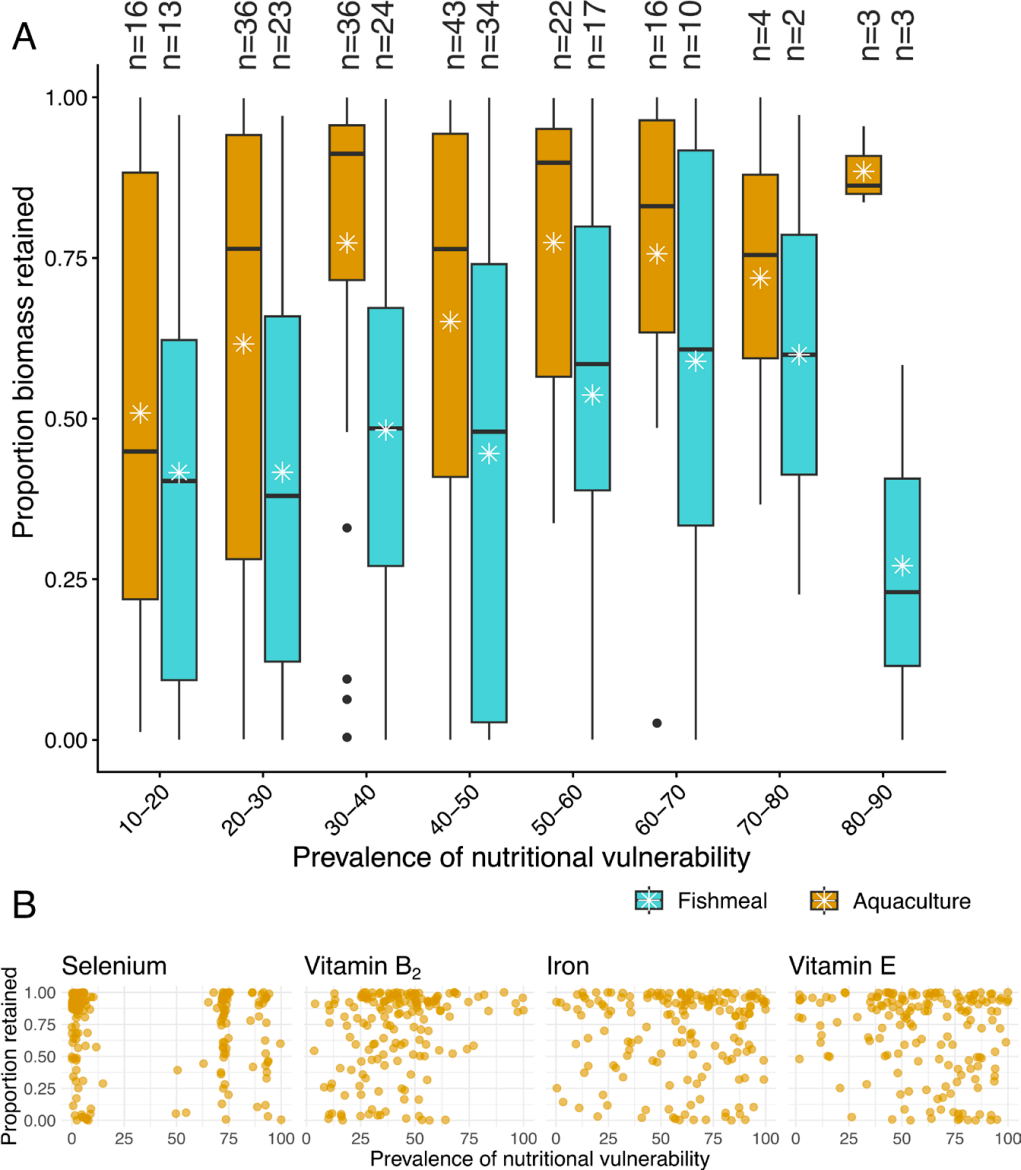
Domestic retention of biomass and nutrients by country nutritional vulnerability. Data are shown for 193 countries and territories between 2015 and 2019. The proportion of countries’ domestically retained biomass of fishmeal (blue) and aquaculture products (yellow) by binned country nutritional vulnerability (*A*). Stars represent averages and points represent outliers. Number of countries per vulnerability group are provided above each box. Proportion retained nutrients from aquaculture products by average percent vulnerable population (*B*). Each point represents a country average. Nutrients in B represent the top 2 nutrients by highest proportion retained (calcium and iron) and the top 2 by highest proportion traded (vitamin A and selenium). Additional nutrients are in *SI Appendix*, Fig. S1.

The majority of edible weight and nutrients from farmed fish and fishmeal were retained within countries (Fig. 1), highlighting the potential role of domestically oriented aquaculture in supporting food security and human nutrition. Retention overall was high (77.7% of edible weight and 76.8% of nutrients). It was highest for freshwater farmed fish (93.3% of edible weight and 94.4% of nutrients), followed by marine farmed fish (62.2% of edible weight and 66.2% of nutrients), and ultimately fishmeal (i.e., 37.1% of potentially edible weight of species converted to fishmeal and 34.1% nutrients). Jointly, these results suggest a potentially high contribution from farmed fish to reducing nutritional vulnerabilities in producer countries. There is a need for subsequent research on subnational distribution to explore whether those most in need benefit from domestically retained farmed aquatic foods, by assessing relative prices, income distribution, waste and loss, and individual consumer preferences (7, 9, 12).

### 1.2. Distributional Equity of Nutrient Trade Flows.

**Fig. 2.**
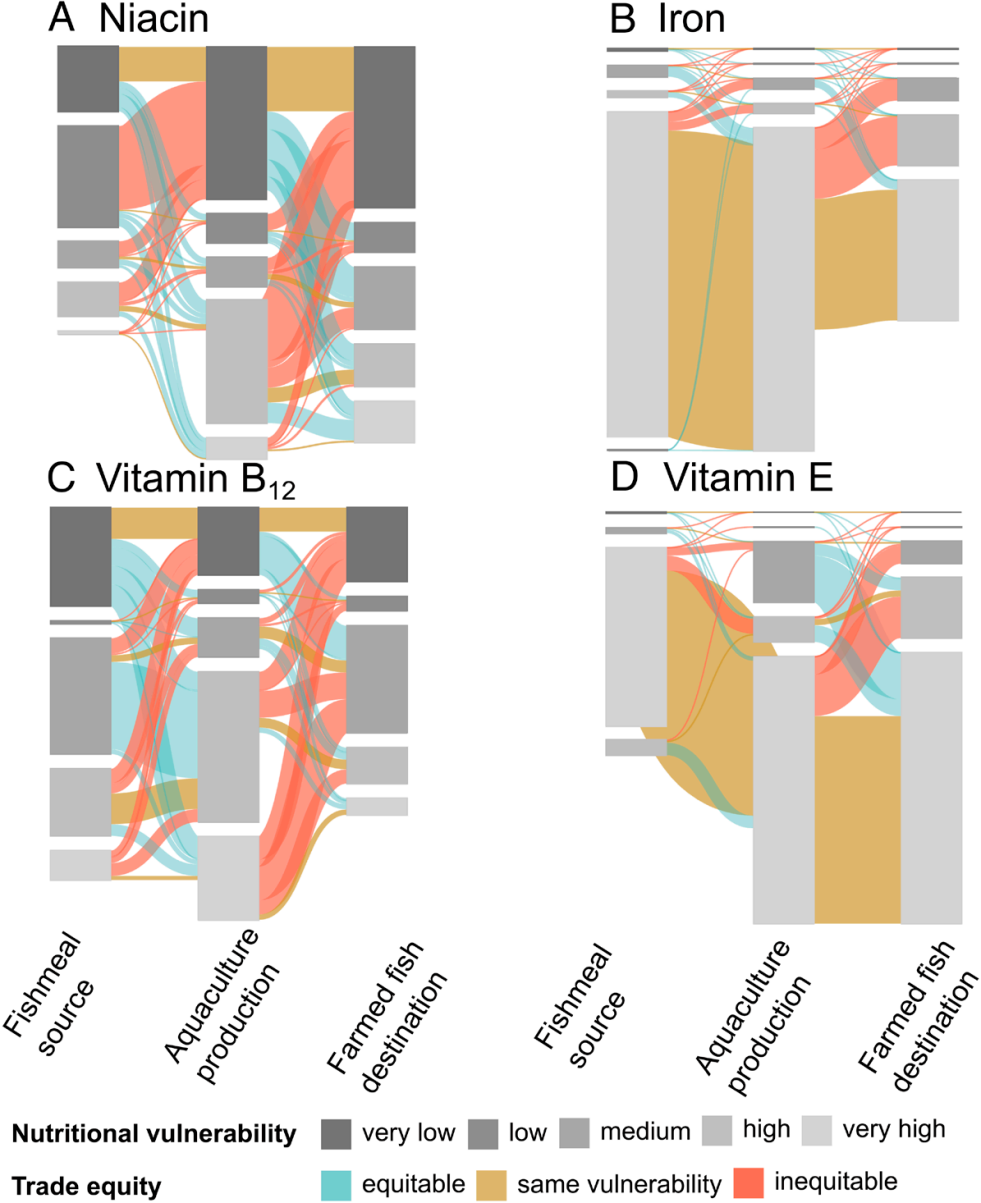
Aquaculture-related trade of niacin (*A*), iron (*B*), vitamin B12 (*C*), and vitamin E (*D*). Niacin and iron were the least and most equitably traded nutrients in fishmeal trade. Vitamins B_12_ and E were the least and most equitably traded nutrients in aquaculture trade. Sankey diagrams represent nutrient trade flows from countries producing fishmeal (left nodes) to aquaculture-producing (middle nodes) and farmed fish-consuming countries (right nodes). Prevalence of nutritional vulnerability is measured by nutrient intake inadequacies and referenced by categories: “very high” countries with >50% of the population with nutrient intake inadequacies; “high,” 25 < % ≤ 50; “medium,” 10 < % ≤ 25; “low,” 5 < % ≤ 10; “very low,” ≤5%. Link color indicates trade flows from countries with higher to lower (red), same (yellow), and lower to higher (blue) intake inadequacy. Link width indicates the trade flow quantities by nutrients (*SI Appendix*, Fig. S4 provides diagrams of the remaining nutrients).

Trade in fishmeal and farmed fish redistributed nutrients from source countries to countries where they were ultimately consumed (Fig. 2). Here, we assessed trade flows by linking countries that produce and export fishmeal to aquaculture-producing countries to countries ultimately consuming farmed fish (Section 3.4). First, we found that traded aquaculture (5.0 million tons edible weight) and supply of fishmeal (3.9 million tons edible weight) were similar, despite vastly different production volumes. From 2015 to 2019, 62.9% of fishmeal production was traded internationally compared to only 14.9% of farmed fish, highlighting the high level of globalization in sourcing fishmeal for aquaculture production.

As in capture fisheries (20), nutrients flowed toward more nutrient-secure nations that import farmed fish. After accounting for production, import, and export, countries with an average net nutrient gain had more adequate nutrient intake (23.5% prevalence of inadequate intake) compared to countries with net nutrient losses (32.6%). These differences in country vulnerability were pronounced for vitamin B_12_, calcium, and vitamin B_2_ (*SI Appendix*, Table S3). Measured in terms of annual individual nutrient needs (median across the studied nutrients), the countries with the largest net gains were the United States (equivalent to the annual nutrient needs of 2.9 million individuals), Japan (1.7 million), and France (738 thousand). The countries with the largest net losses were Peru (5.7 million), Chile (3.2 million), and Norway (3.2 million). There was strong correspondence across scenarios, with the same set of countries appearing in the top total gains and losses (*SI Appendix*, Table S4), except reversed ranks when accounting for fishmeal containing 34% of trimmings used in 2022 (4) and Korea, China, and Vietnam appearing in the third largest losses in calcium, iron, and vitamin E, respectively. This indicates that global gains and losses are driven mainly by traded weights rather than by nutrient variation across species portfolios.

On the supply side, most traded farmed fish stem from vulnerable regions. The majority of all nutrients were exported from nutritionally vulnerable countries (defined as high >25% or very high >50% inadequate nutrient intake), though less so for the traded weight of fishmeal (57.7%) than for farmed fish (66.3%). While this pattern (i.e., fish stem from vulnerable regions) was robust to varying vulnerabilities, the total proportion of trade flows from vulnerable countries was sensitive to vulnerability thresholds (varying for fishmeal between 55.2 and 62.0% and for aquaculture between 62.9 and 74.5%; *SI Appendix*, Fig. S2).

The loss of nutrients from nutritionally vulnerable countries does not necessarily translate into increased nutritional vulnerability. While we found that a large proportion of nutritionally vulnerable countries lost nutrients in farmed fish and fishmeal through trade (44.7% of countries with >50% prevalence of inadequate intake), economic variables can interact with nutrient loss. For example, where exports of high-value aquatic foods are replaced by imports of affordable aquatic foods (6), nutritional vulnerability may be reduced despite nutrient loss from trade. Future assessments could focus on supply chain characteristics, links between earnings and improved nutrient availability from alternative food sources, and improved affordability related to imported aquatic foods.

Trade in farmed fish was more inequitable than trade in fishmeal for most nutrients [i.e., nutrient exports from more to less vulnerable countries were higher for aquaculture (36.5%) than for fishmeal (20.6%; Fig. 2)]. Using absolute values instead of vulnerability categories showed the same pattern but with higher estimates of inequitable trade (46.0% for aquaculture and 30.0% for fishmeal; *SI Appendix*, Table S5). Nutritional equity in trade varied among nutrients (Fig. 2 and *SI Appendix*, Fig. S3). For example, vitamin B_12,_ contained in traded farmed fish, was primarily exported from countries categorized as having high and very high nutritional vulnerability to countries with very low and medium nutritional vulnerability (Fig. 2*C*). Impacts on the supply of vitamin B_12_ are of substantial concern due to the high global prevalence of suboptimal B_12_ intake (26.9% on average across countries), and it is linked to a variety of undesirable outcomes, particularly in children, where it can stunt brain and intellectual development (29). In contrast, iron contained in fishmeal trade flows was categorized as equitable, primarily because countries that export fishmeal and countries that import fishmeal were both categorized as highly vulnerable to inadequate intake of iron (Fig. 2*B*). Yet, final farmed fish destinations had frequently high and medium nutritional vulnerability, indicating that important iron supply is channeled away from those in need. Iron deficiency is associated with cognitive impairment and severe anemia, and the global prevalence of iron deficiency anemia in children under five is >16% (30).

### 1.3. Distributional Equity of Nutrient Trade Flows by Aquaculture Taxa.

**Fig. 3.**
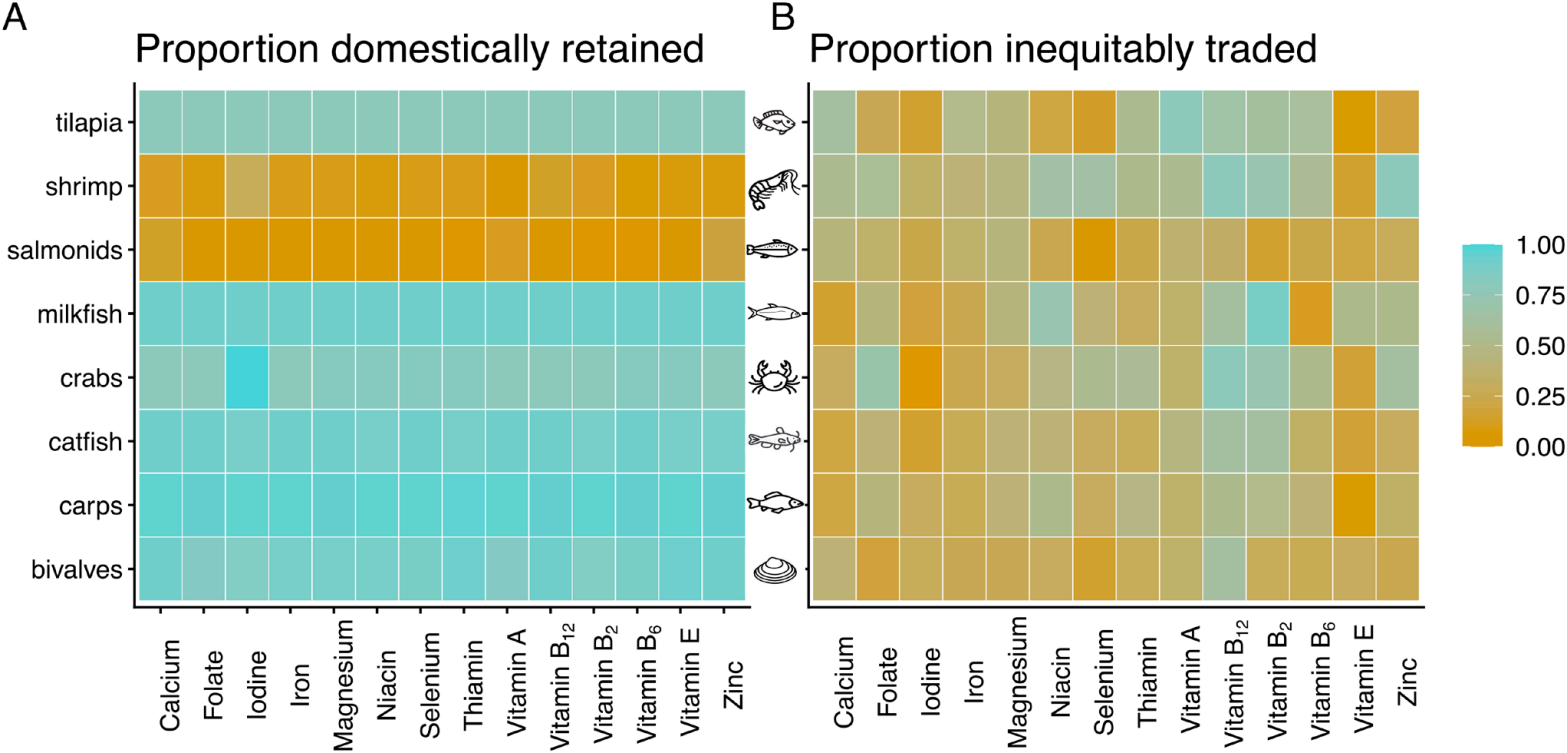
Domestic retention and trade inequity in farmed fish trade flows. The *Left* represents the proportion of nutrients retained domestically (*A*), and the *Right* shows the proportion of traded volumes from more to less vulnerable countries by taxa and nutrients (*B*). Values have been averaged across trade partners. *SI Appendix*, Figs. S5 and S6 provide the same heatmaps for fishmeal trade and across different country vulnerability thresholds.

Aquaculture production represents a diverse set of taxa that differ in their market destinations. Therefore, as above (Section 1.2), we compared equity in trade flows across individual aquaculture taxa. We linked fishmeal trade flows to the production of aquaculture taxa by calculating total demand from feed conversion ratios and feed composition values [Section 3.3; (31)]. Domestic retention was high across most taxa in farmed fish product trade (83.6% for tilapia and higher for other taxa), except for shrimp and salmonids (Fig. 3*A*). Salmonids and shrimp are both high-value taxa and require substantial albeit declining fishmeal input in production (5). They also account for a high proportion of global aquatic food trade value [37% of total value in 2022; (4)]. Yet, they were at opposite ends of the trade equity spectrum (Fig. 3*B*). Trade in shrimp and crabs (55.4% and 45.5%) was almost twice as inequitable as trade in salmonids and bivalves (28.1% and 30.7%). While price may be a primary driver of variability in retention across taxa, our results suggest it is less suited to explaining patterns of nutritional inequity.

**Fig. 4.**
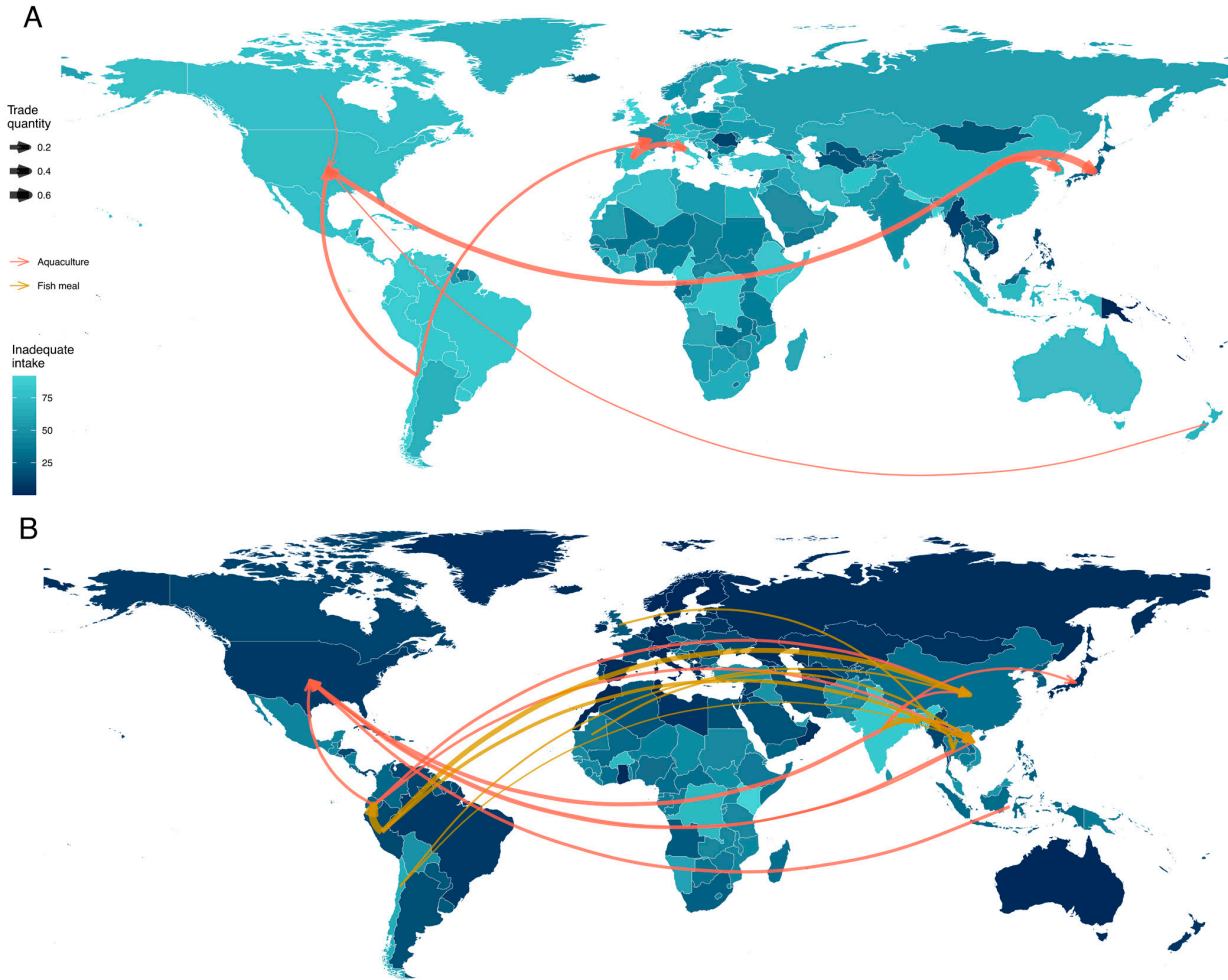
Top 10 trade flows by edible weight of selected taxa–nutrient pairs. Vitamin E in traded bivalve network (*A*) and vitamin B_12_ in traded shrimp network (*B*) represent lower and upper ends of inequitable trade, respectively. Country colors represent the proportion of the population with inadequate nutrient intake from low (green) to high (blue). The trade flows represented by arrows in the figures account for nearly half of total traded weight in taxa (48.2% in bivalves; 44.1% in shrimp). The color of arrows represent fishmeal (yellow) and farmed fish trade (red). Increasing arrow width represents higher amounts of total traded nutrients. No fishmeal trade flows are represented in bivalves which belong to non-fed aquaculture taxa.

Trade of specific nutrient and taxa pairs in aquaculture-related trade networks showed distinct variability in the equity of trade flows and country-level vulnerabilities (Fig. 4). Bivalves (vitamin E) and shrimp (vitamin B_12_) illustrate the differences at the lower and higher ends of inequitable trade, respectively. From all trade in fishmeal and farmed fish, bivalve aquaculture trade flows accounted for 5.0%, and shrimp for 23.4% of the total traded aquaculture edible weight. A few species dominated trade in both groups. Chilean mussels (*Mytilus chilensis*) represented about one-fifth (17.8%) of traded bivalve edible weight. They were exported from Chile primarily to the United States, France, Italy, and Russia (jointly accounting for 57.0% of imports). Other notable taxa included mussels (*Mytilidae*) and the Manila clam (*Ruditapes philippinarum*); 45.4% of trade was composed of these three taxa. Equitable trade in bivalves was linked to high vitamin E vulnerability across importer and exporter countries, with all but one importer (Japan) in the top 10 trades by weight having very high inadequate intake (Fig. 4*A*). In shrimp aquaculture, white leg shrimp (*Litopenaeus vannamei*) accounted for more than three-quarters (81.2%) of the total edible weight, and more than half (53.3%) was produced in India and Ecuador. It was also the most traded species, with India and Ecuador accounting for nearly two-thirds of exports (63.7%) and the United States for nearly one-third of imports (31.7%). Inequitable trade in the shrimp trade network was linked to high nutrient exports from nutritionally vulnerable countries. All 10 of the largest exporters had high or very high vitamin B_12_ vulnerability (Fig. 4*B*).

### 1.4. Potential and Actual Nutritional Benefits from Retaining and Trading Fishmeal and Farmed Fish.

To understand whether aquaculture trade affects nutritional vulnerability, we assessed nutrient trade alongside the nutritional vulnerability of source and destination countries. We tested a hypothetical scenario of repurposing and domestically retaining aquatic foods. We found that aquaculture source countries (46.6%; n = 164) were somewhat more vulnerable than destination countries (40.6%; n = 192). We also found, on average across years and nutrients, that aquaculture imports in destination countries could have newly met the nutrient needs of 45.5 million individuals with inadequate intake. If farmed fish had been retained for direct human consumption, they could have met the nutrient needs of an additional 36.1 million people who currently have inadequate nutrient intake. Similarly, if captured aquatic species used for fishmeal had been consumed directly, they could have met the needs of 31.0 million people (or 23.3 million if fishmeal from trimmings was reduced by 34%).

Average nutritional vulnerability in exporting countries was higher than in importing countries. Yet, farmed fish imports, compared with hypothetical retention and consumption of exported fishmeal or farmed fish, could potentially make the highest contribution to nutritional needs. These seemingly contradictory results (i.e., higher vulnerability in exporting countries alongside higher nutritional benefits in importing countries) are explained by the structure of aquaculture trade. A few large exporters dominate aquaculture exports, whereas imports are dispersed, serving a larger number of countries that import small volumes. For instance, nearly three times as many importers (n = 16) account for nearly three-quarters (74.8%) of total traded farmed fish weight compared to exporters (n = 6, 72.0%). Whether nutrient distribution is more equal in the presence of trade (e.g., ref. 23) thus depends on the equity of domestic distribution, i.e., assuming that nutrients (in comparatively nutrient-rich aquaculture importing countries) were distributed to those in need.

We tested hypothetical scenarios of repurposing and domestically retaining farmed fish. We captured aquatic food used as fishmeal input, assuming that appropriate domestic consumer demand existed and all captured aquatic food could be used for human consumption. Repurposing captured aquatic food in fishmeal production and reducing food loss and waste have been estimated to double the nutrients from aquatic foods available today (32). Yet, many marine and terrestrial plants and animals fuel the rapid and sustained growth in aquaculture. Therefore, aquaculture’s contribution to global nutrition relies on its dynamic interaction and embeddedness within the food system (16). For instance, aquaculture may impede by-catch reduction due to high feed prices (33) or compete with the development of edible products from trimmings (34), thereby interacting with food availability more broadly. Beyond fishmeal, other marine and terrestrial feed ingredients, from domestic production or imported, are also used in fish farming, which limits their potential to contribute to human nutritional outcomes (16).

**Fig. 5.**
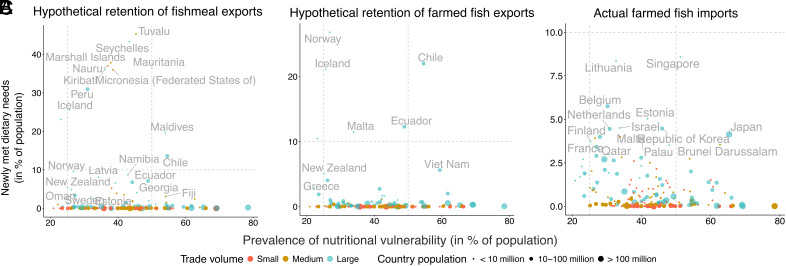
Proportion of the nutritionally vulnerable population by country and proportion of the population that could meet individual nutrient needs (i.e., the average number of people with inadequate nutrient intake that could meet annual nutrient recommendations of 14 nutrients) by repurposing aquatic foods from wild fish used in fishmeal production and domestically retaining wild and farmed fish. (*A*) represents hypothetically repurposing and retaining fish used in the production of fishmeal exports; (*B*) represents hypothetically retaining farmed fish exports for domestic consumption; (*C*) represents actual farmed fish imports. Color represents trade volumes separated into tertiles. Circle size represents countries’ total population. For countries where the total number of newly met individual nutrient needs is larger than the nutritionally vulnerable population, values were limited to 100 percent.

Our scenarios show that countries could have more potential to reduce inadequate nutrient intake levels by retaining and repurposing wild-caught fish used for fishmeal in comparison to retaining or importing farmed fish products. Repurposing captured aquatic food which is currently used for feed could nearly eliminate inadequate nutrient intake in eight countries (i.e., Tuvalu, Seychelles, Marshall Islands, Nauru, Kiribati, Peru, Iceland, and Denmark). Additionally, for a dozen countries, repurposing captured aquatic food used in fishmeal could have met the nutritional needs of more than 10% of their total populations (Fig. 5). There was a smaller effect on reducing inadequate intake when considering reductions in fishmeal volume from trimmings (i.e., three countries could eliminate inadequate nutrient intake, and eleven countries with more than 10% newly met dietary needs). From farmed fish trade, six exporting countries (from hypothetical retention) and zero importing countries could potentially meet the nutritional needs of more than 10% of their populations (Fig. 5 *B* and *C*). This pattern—most countries meeting the nutritional needs of their total populations from hypothetical repurposing aquatic food currently used for fishmeal and a few countries from actual farmed fish imports—was consistent for potentially newly meeting the nutritional needs of more than 3, 5, and 10% of their populations (*SI Appendix*, Fig. S7). This finding is central to assessing trade benefits because of the substantial trade-off between using aquatic food for direct human consumption and for aquaculture feed.

Small-island, South American, and West African countries were highlighted in our hypothetical scenarios of repurposing captured aquatic food and retaining farmed fish (Fig. 5 *A* and *B*). Estimating the full set of nutritional benefits these countries could ultimately attain requires understanding the broader food system in which retaining and repurposing would occur. On the one hand, economic benefits from exports may outweigh nutritional benefits when benefit-sharing mechanisms, such as preferential arrangements for small-scale producers, are in place (35) or when production exceeds demand (e.g., excessive catch volumes in Peru). In these cases, trade may contribute to dietary diversity beyond aquatic foods (e.g., ref. 36), though this may mean replacing aquatic foods with nutrient-poor, energy-dense foods (e.g., ref. 37). On the other hand, nutritional benefits are likely to accrue where fishmeal and aquaculture production and export affect fish consumption (38, 39) and reduce the availability of nutrient-rich pelagic fish (20, 40). For example, in Senegal and Mauritania, where fishmeal exports were jointly equivalent to 1.8 million individual annual nutrient needs, processing plants are reported to target traditionally consumed sardinella species (39), contributing to halve fish consumption in Senegal in the past two decades (41). Thus, future work aiming to enhance nutritional resilience and equity through repurposing and retention must take into account the various cultural, social, and economic dimensions associated with individual aquatic foods within broader food systems.

An important extension, building on our work, will have to account for countries’ demographics in evaluating nutritional equity. Nutritional vulnerabilities change with life stages and differ by biological sex. For instance, removing nutrients from a country with a high prevalence of inadequate intake in children or pregnant women differs from a country where inadequate intake is primarily prevalent in middle-aged men. Quantifying these factors must be based on a comprehensive framework to evaluate the disproportionate health burden on certain groups.

## 2. Conclusion

Aquaculture’s contribution to domestic nutrition worldwide, for several nutrients that are important to human health, was potentially high, but trade disproportionately disadvantaged nutrient-vulnerable countries. Current food systems still fall short in providing equitable, sustainable, and just nutritional benefits (42), with continued malnutrition occurring globally, especially in women and children (4, 26). The expansion of aquaculture holds the promise of playing a central role in achieving key SDGs, such as Goal 2: Zero Hunger and Goal 14: Life Below Water (3). Our analysis demonstrated value in domestic retention of captured aquatic food used for fishmeal export. It also highlighted equitable trade of farmed fish products as one potential intervention point for policies aimed at sustainable and healthy food systems. For instance, repurposing one-third of wild fish used in fishmeal production for direct human consumption could increase nutrient availability, while maintaining by-products for use in aquaculture feeds (43). Sustainable fisheries management and aquaculture development are other potential solutions to enhance the contribution of aquaculture systems—and aquatic foods more broadly—to global nutrition (44). Yet, production and trade policies are often centered around revenue growth and economic optimization (45). While foreign exchange earnings and economic variables are important, they alone will not ensure that aquatic foods reach the most nutritionally vulnerable (13, 46). Specifically tailoring food and trade policies in a concerted effort could thus enhance the nutritional outcomes of aquaculture (47, 48). Ultimately, aquaculture production and trade should therefore form part of an integrated approach that rethinks existing policies with human health and nutrition as their primary objectives.

## 3. Materials and Methods

Our study examines the distributional equity of aquaculture-related nutrient production and international trade. We focused on domestic use, trade, and nutrient composition of fishmeal and aquaculture animal products for 14 nutrients for human health (27). We converted all values to edible proportions. Acknowledging that edible proportions vary by cultural context, processing efficiency, and species anatomy, we used average muscle fractions from live weight (31). We used the average nutrient concentration of muscle tissue as a representative value of fish and aquatic invertebrates’ nutrient value. This means edible proportion and nutrient content from muscle tissue overlap. All presented values, unless otherwise indicated, are averages from 2015 to 2019. We averaged data from the most recent 5 y available, excluding the COVID pandemic years, to provide an up-to-date overview of the aquaculture sector. First, we merged the ARTIS and AFCD databases (Section 3.1 and *SI Appendix*, Fig. S8) to estimate total nutrient supply, domestic retention, and the traded proportion of nutrients from aquaculture. Second, we combined data on countries’ vulnerability for specific nutrients (Section 3.2) to assess the equity of nutrient distribution in farmed fish and fishmeal trade flows from or to nutritionally vulnerable countries. Third, in our analysis, we explored the potential nutrient contributions from aquatic foods through two scenarios: i) hypothetically retaining wild fish in producing countries that would otherwise be exported as fishmeal, and ii) hypothetically retaining farmed fish in producing countries that would otherwise be exported, comparing these to actual farmed fish imports in importing countries (Section 3.5). We only focused on the additional nutrient intake from this retention; other nutrient intake sources (e.g., portion of production that is domestically consumed, actual imports) are not indicated in the analysis. The supplementary information provides a complete overview of the databases used in the analysis (*SI Appendix*, Table S6).

### 3.1. Trade and Nutrition Data.

The AFCD and ARTIS databases combined provide a central component to studying nutrient production and distribution from farmed fish. The AFCD combines aquatic food composition data from international and national food composition tables, as well as peer-reviewed data; it totals 29,912 observations and 3,753 unique taxa (25). The database includes taxonomic levels (species to kingdom), edible proportion coefficients, and 374 micronutrient values. We selected 14 nutrients necessary for human health: six minerals (calcium, iodine, iron, magnesium, selenium, and zinc) and eight vitamins [A, B_1_ (thiamin), B_2_ (riboflavin), B_3_ (niacin), B_6_, B_9_ (folate), and B_12_ (cobalamin), E]. We used Gephart et al.’s edible proportions, representing average muscle fraction from live weight for 22 farmed and wild capture species groups (31). Where edible proportion coefficients were not available (25), we imputed them to match the edible proportion values of the next available taxonomic level. This means if a species had no species-specific edible proportions in the data, we assigned that value. If no value was available at the species level, we assigned the mean of the next taxonomic level (e.g., genus). The resulting taxonomic resolution is reported in *SI Appendix*, Fig. S7. Averaging across multiple entries reduced taxonomic resolution but also contributed to balancing cultural and processing-specific values from single observations. Nutrient content is reported by unit (i.e., mg or μg) per 100 g of live weight (converted to tons for further analysis). Outliers were defined as nutrient values larger than three times the interquartile range. Data points lying above or below these thresholds were removed from the dataset. Nutrient composition represents the nutrient content across all selected nutrients of a given species. We used the maximum available nutrient composition data by averaging reported nutrient values of species and higher taxonomic levels, such as genus and family, across studies. We assumed that the raw muscle tissue nutrient concentration was representative of the edible weight. For consistency, we chose not to combine different preparation types and body parts. Food preparation type “raw” (56.8%) and body parts “muscle tissue” (53.2%) featured the highest number of samples in AFCD. Muscle tissue nutrient concentrations are typically lower for vitamins A, B_12_, and E, and minerals such as iron and zinc than for other body parts. Therefore, the statistics presented in our analysis represent a conservative estimate. The AFCD provides limited data on fished versus farmed species; therefore, we used the same average nutrient composition for all species.

The ARTIS database represents the first publicly available database of aquatic resources trade at the species level. It provides the apparent consumption (total supply from production and imports minus exports) of live weights for all farmed and wild aquatic species from 1996 to 2020 (24). It represents a disaggregation of reported aquatic product trade into species or species groups based on a mass balance model that accounts for re-exports. The entire database consists of >2,400 species groups, 193 countries, and more than 35 million bilateral trade records. Complex aquatic food supply chains with multiple export steps complicate the analysis; therefore, we improved the accuracy of apparent consumption estimates by accounting for re-exports. Note that ARTIS traces trade back to the producing country, which does not necessarily represent the geographic location of a country’s catches in the case of distant water fishing. Also, ARTIS disaggregates traded products to species group level, but is not explicitly designed to estimate the end use of production (e.g., human direct intake, fishmeal) for domestically produced and retained products (24). The subset of data used in our analysis contains source country (exporter), destination country (importer), scientific name of species, production method, and live weight information from 2015 to 2019. It excludes fish oil, aquatic plants, and algae. Despite the importance of fish oil in feeds (as well as nutraceutical and other uses), we did not explicitly account for it due to double accounting when fishmeal and fish oil are produced from the same product. We removed all fishmeal sourced from aquaculture production, as it primarily comes from by-products. We also removed observations that were not associated with a producing country (4% of total weight). These observations represent exports that cannot be explained by the reported production and import data alone. They indicate underreported catch, underreported imports, or issues with the live weight conversion factors (i.e., conversion from live weight to fishmeal).

To jointly analyze global trade flows and composition of different kinds of aquatic foods, we linked the databases by species or the next available taxonomic level up to phylum (taxonomic levels of matches in *SI Appendix*, Fig. S7). Our analysis simplifies nutrient flows from fish trade by focusing on the nutrient content of muscle tissue and fishmeal for feed. Still, it does not account for other traded products—such as whole fish, fish parts, or nutraceuticals—which also contribute to nutrient flows.

Unless otherwise specified, we converted all combined data from reported live weight values to edible weight using edible proportion conversion factors (23). Nutrient supply was calculated as a function of edible weight and nutrient composition. We converted daily RDAs to annual values to match bilateral trade data using 365 d as a multiplier. Where indicated, we normalized across nutrients by per capita nutrient RDAs (Section 3.2), so that nutrient values presented in the analysis are expressed in annual individual dietary intake needs.

### 3.2. Nutrient Needs and Country-Level Nutritional Vulnerability.

To estimate potential nutrient intake from apparent consumption of aquatic foods, we converted nutrient supply into nutrient-specific requirements to normalize each nutrient to the same unit. We calculated annual nutrient needs for our study by multiplying the recommended dietary allowances (RDAs) for the 14 key nutrients in our study by the number of days in the year (49). We used dietary allowances for women aged 14 to 50 for our analysis. These values are slightly above those of men in the same age category. We converted edible weight to total nutrient supply. We divided the supply by the annual RDAs to calculate the number of individual nutritional needs produced by a country’s aquaculture production for each focal nutrient. RDAs represent the nutrient requirements of most healthy individuals of particular age–sex groups and thus overestimate population-level requirements (49).

Individual nutrient needs should not be met exclusively through aquatic foods. Yet, calculating the number of individuals whose nutritional needs could potentially be met allowed us to assess the theoretical impact of aquaculture production and trade. In the newly approved minimum dietary diversity indicator for women of reproductive age for tracking progress on SDG2, a minimum of five food groups should be consumed per day (28). However, the indicator does not quantify the consumption of total amounts of biomass or nutrients from each of these food groups that could provide a robust basis for calculating the aquatic food contribution to diet. This means, in addition to estimating met nutrient requirements by using edible instead of live weight, muscle tissue nutrient concentrations (lower than, for example, in viscera), and above-average individual nutrient needs (RDAs overestimate population-level requirements), the statistics presented in this article provide highly conservative estimates of nutrient contribution.

Country nutritional vulnerability is represented by the prevalence of inadequate intakes (44). Passarelli et al. used census data from 2018 to calculate population-level inadequate intake values. FAO collects a wide range of food security indicators that are widely used (49). The indicator most related to our analysis is the prevalence of undernourishment. For all nutrients and most countries, the average inadequate intake is higher than the prevalence of undernourishment (*SI Appendix*, Fig. S9). Particularly high is the difference between inadequate intake of calcium, iron, iodine, vitamin E, and zinc and the prevalence of undernourishment. Following Nash et al. (20), we categorized countries’ nutritional vulnerability: very high countries with >50% of the population with inadequate nutrient intake; high, 25 < % ≤ 50; medium, 10 < % ≤ 25; low, 5 < % ≤ 10; very low, ≤5% (*SI Appendix*, Table S7). Values representing multiple nutrients are all trade volume-weighted averages. We categorized countries as nutritionally vulnerable if they have high or very high inadequate nutrient intake. Finally, we tested the sensitivity of countries’ nutritional vulnerability by varying cut-off values (*SI Appendix*, Table S8). Despite the role of omega-3 and omega-6 fatty acids from aquatic foods in diets, we were not able to integrate them in this analysis due to a lack of reliable global country-level data on inadequate intake of these polyunsaturated fatty acids to match the remainder of our analysis.

### 3.3. Fishmeal Consumption Estimates.

We estimated fishmeal consumption volumes of aquaculture production by taxa and by country from trade data and feed conversion ratios for the result Section 1.3. Due to a lack of global scale data linking fishmeal by origin and species to aquaculture production, we estimated fishmeal use for individual taxa produced in aquaculture using a bottom–up approach. The bottom–up approach estimates fishmeal consumption used in ARTIS aquaculture production volumes, feed conversion ratios, and the proportion of fishmeal and oil in feed. First, we used International Standard Statistical Classification of Aquatic Animals and Plants (ISSCAAP) groups to match feed conversion ratios and the feed composition database with species from ARTIS (details below). Second, we calculated fishmeal consumption volume as a function of

$$FM=\sum_{0}^{s=T}AC_{s}xFCR_{s}xP_{\mathrm{FMFO}}xLWC_{\mathrm{FM}},$$

where, fishmeal denoted by FM and aquaculture denoted by AC live weight are given in tons, *s* represents the species group, FCR the feed conversion ratio of a given species, *P_FMFO_* the proportion of fishmeal and fish oil in feed composition, and *LWC_FM_* the live weight conversion factors of 2.98 of fishmeal (i.e. conversion from live weight to fishmeal accounting for trimmings) used as a constant across species (50). Due to the *P_FMFO_* value, the estimate contains fish oil, which is not included in other parts of the analysis.

FCR and feed composition values were derived from the Monterey Bay Aquarium’s Seafood Watch program, farm-level data collected for a range of systems in Southeast Asia, and published life-cycle analyses (29). Multiple taxonomic levels were present in both ISSCAAP Groups and FCR data. The FCR database contains 12 aquatic taxa, such as bivalves, salmon, and milkfish. We matched taxa if i) FCR taxa were mentioned explicitly in the ISSCAAP group, ii) if FCR taxa fitted into the broader ISSCAAP group, and iii) if the ISSCAAP group entirely (all mentioned species) fitted into the broader FCR taxa. We would not match an ISSCAAP group to a broader taxa category that already had more specific taxa matched. This means multiple taxa from the FCR database could be matched to one ISSCAAP group, and multiple ISSCAAP groups could be matched to one taxon from the FCR database. To match the higher aggregate taxa, we calculated average FCR values.

After having calculated total fishmeal demand, we aimed to assign fishmeal trade flows of species and countries to aquaculture production. First, to identify species and their nutrient composition, we assumed that the species composition going into fishmeal was the same for all farmed fish taxa. We also assumed that aquaculture producers sourced fishmeal for specific taxa from the same countries from which they sourced all their fishmeal. This means we retain the composition of trade partners and the proportion of their trade to total imports, but scale them to the fishmeal needed to produce a single aquaculture taxon. In addition, we retain the composition of fishmeal species and the proportion of their trade to total imports and scale them to the fishmeal needed to produce a single aquaculture taxon in a given country. This approach has two limitations: it excludes fishmeal-consuming countries without aquaculture production from the data, and it likely overestimates the number of species and importers involved in the production of individual aquaculture species.

### 3.4. Estimating Equity of Trade Flows.

To assess the equity of aquaculture products and fishmeal nutrient trade flows, we linked fishmeal exports and imports to bilateral farmed fish trade and apparent consumption data. First, we linked the bilateral trade flows at the country-level, i.e., fishmeal importers were matched to aquaculture producers. Nutrient supply from trade flows was calculated as described above (Section 3.1). Then, we classified countries from very low to very high nutritionally vulnerable as measured by the prevalence of inadequate nutrient intake for specified nutrients (Section 3.2).

Using vulnerability and trade data, we calculated statistics on the source and destination of products and the nutritional differences of trade partners. First, we calculated the domestically retained and traded portion of production by total weight and nutrient supply from fishmeal and aquaculture products. Products are represented in ARTIS independent of whether they stayed in-country, were exported, or re-exported.

In the next step, we removed the domestic retained portion and exclusively assessed trade flows in the data (Sections 1.2–1.4). We calculated nutrient supply per nutrient and used nutrient median values to classify countries as nutritionally vulnerable (Section 3.2). Finally, we calculated the proportion of inequitably traded nutrients from fishmeal and aquaculture products. Trades between countries of the same vulnerability classification, i.e., medium to medium nutritionally vulnerable, were classified as equitable. We also categorized trades as equitable if they flowed from more to less nutritionally vulnerable countries, but as inequitable in the opposite direction, i.e., from less to more nutritionally vulnerable countries. Variations in equity of trade flows between nutrients were caused by differences in species’ nutrient content and country vulnerability levels for individual nutrients.

Finally, we alternatively tested calculating the proportion of inequitable trade flows using absolute numbers. We classified trade as

$$\begin{array}{c} \mathrm{Trade}\;\mathrm{equity}\;\mathrm{categories}\}\begin{array}{cc} \mathrm{equitable}, & \mathrm{if}I_{D}+5\%>I_{s}\\ \mathrm{trade}\;\mathrm{with}\;\mathrm{same}\;\mathrm{vulnerability}, & \mathrm{if}I_{D}-I_{s}\leq |5\%|\\ \mathrm{inequitable}, & \mathrm{if}I_{D}<I_{s}+5\% \end{array}, \end{array}$$

where *I_D_* and *I_S_* represent inadequate intake of the destination and source countries. We provided these additional values for fishmeal and aquaculture products in *SI Appendix*, Table S5.

### 3.5. Trading and Retaining Nutrients from Fishmeal and Aquaculture.

We tested a hypothetical scenario to compare the potential nutritional benefits of domestically retaining and repurposing fish and invertebrates used in fishmeal and farmed fish exports and actual farmed fish imports. We calculated nutrient supply for fishmeal-exporting, aquaculture-exporting, and aquaculture-importing countries. A country may fall into all of these categories if it exports and imports fishmeal and farmed fish. Nutrient supply from exports and imports was calculated as described above (Section 3.1).

We calculated the total global number of individuals and the average proportion of a country’s population newly meeting annual dietary needs measured by RDAs from the nutrient supply of 14 nutrients from domestically retained aquaculture products and repurposed fish and invertebrates used in the production of fishmeal. Countries’ proportion of newly met individual nutrient needs could not be larger than the proportion of the total nutritionally vulnerable population (Section 3.2). Then, we compared these values to potential contributions from actual aquaculture imports. We also calculated net nutrient gains and losses by subtracting the number of individual nutrient needs from fishmeal and aquaculture exports from the number of individual nutrient needs from aquaculture imports.

In calculating the hypothetical scenarios of potential nutritional benefits from domestic retention and repurposing, we assumed that products could be directly consumed by humans. Most farmed fish products are for direct human consumption. However, only a small proportion of fishmeal is reported as fit for human consumption, despite the majority of the fish used to produce fishmeal being food grade (17), with 0,2% of total fishmeal production in the FAO Processed Product data being reported as commodity code “fishmeal fit for human consumption, nei” (51).

### 3.6. Fishmeal Sourcing Sensitivity.

Fishmeal increasingly originates from sources outside of reduction fisheries, including fishmeal from trimmings, waste, and aquaculture by-products (52, 53). The trade data do not differentiate between fishmeal products sourced from reduction fisheries and sourced from trimmings, except for aquaculture by-products. This is in part due to conflicting global data on sources used in the production of fishmeal. First, in the FAO Processed Product data [commodity code “fishmeal obtained from fish waste/offals”; (49)], the proportion of fishmeal from waste and offals is very low (0.3% of total). However, the FAO reports 34% of fishmeal stems from trimmings, a value that is based on IFFO estimates (4).

Sourcing of fishmeal interacts with our analysis in Sections 1.1 and 1.4. We expect country-level variability in fishmeal sourcing to marginally alter estimates of countries’ gains and losses, which are driven primarily by the larger farmed fish product volumes. We removed fishmeal originating from aquaculture (likely in the form of by-products), thus our baseline calculations assumed all remaining fishmeal was from reduction fisheries. We also tested the sensitivity of our results to changes in the proportion of fishmeal from trimmings (i.e., from 0 to 34%) and reported these alongside the baseline results (Sections 1.1 and 1.4). At the global level, we expect our baseline calculations to generate supply overestimates, whereas the 34% fishmeal from the trimmings scenario, after removing aquaculture by-products, underestimates fishmeal values.

In Section 1.4, we estimated the potential impact of domestic retention and repurposing of fishmeal of various aquatic foods for direct human consumption on nutrient vulnerability. We calculated this hypothetical scenario with the baseline and trimming scenario. Fishmeal from trimmings could potentially be repurposed for direct human consumption; therefore, we have included it in our hypothetical analysis.

## Supplementary Material

Appendix 01 (PDF)

## Data Availability

Previously published data were used for this work. [The ARTIS database is archived on Zenodo (https://doi.org/10.5281/zenodo.10034319) (24) and is publicly available. The AFCD database is archived on Harvard University Dataverse (https://dataverse.harvard.edu/dataverse/afcd) (25) and is publicly available. Links and references to other data are included in *SI Appendix*, Table S6. Code used in preparation of the analysis of this article is available on GitHub (https://github.com/Seafood-Globalization-Lab/fmfo_nutrient_flows.git) (54).]
