# Supplementary material for: Global nutritional equity of fishmeal and aquaculture trade flows
Source: Proc Natl Acad Sci U S A. 2026 Feb 9;123(7):e2506699123. doi: 10.1073/pnas.2506699123 (PMC12912983; doi:10.1073/pnas.2506699123)
Supplement: Supplementary file 1 — Appendix 01 (PDF) [file pnas.2506699123.sapp.pdf]

PNAS

Supporting Information for

Global nutritional equity of fishmeal and aquaculture trade flows

Authors: Laura G. Elsler[1,2], Jessica A. Gephart[3], Jessica Zamborain-Mason[1,2], Tim Cashion[4], Max Troell[5,6], Rosamond L. Naylor[7], Rahul Agrawal Bejarano[3], Christopher D . Golden[1,2]

Affiliations:

1. Department of Nutrition, Harvard T. H. Chan School of Public Health, Boston, MA, USA.
2. Department of Environmental Health, Harvard T. H. Chan School of Public Health, Boston, MA, USA.
3. School of Aquatic and Fishery Sciences, University of Washington, Seattle, WA, USA
4. No Affiliation
5. The Beijer Institute, Royal Swedish Academy of Sciences, Stockholm, Sweden
6. Stockholm Resilience Centre, Stockholm University, Stockholm, Sweden
7. Department of Environmental Social Sciences, Stanford Doerr School of Sustainability, 473 Via Ortega #363, Stanford University, Stanford, CA 94305 USA

Corresponding author: Laura G. Elsler

Email: lelsler@hsph.harvard.edu

This PDF file includes:

Figures S1 to S9
Tables S1 to S8

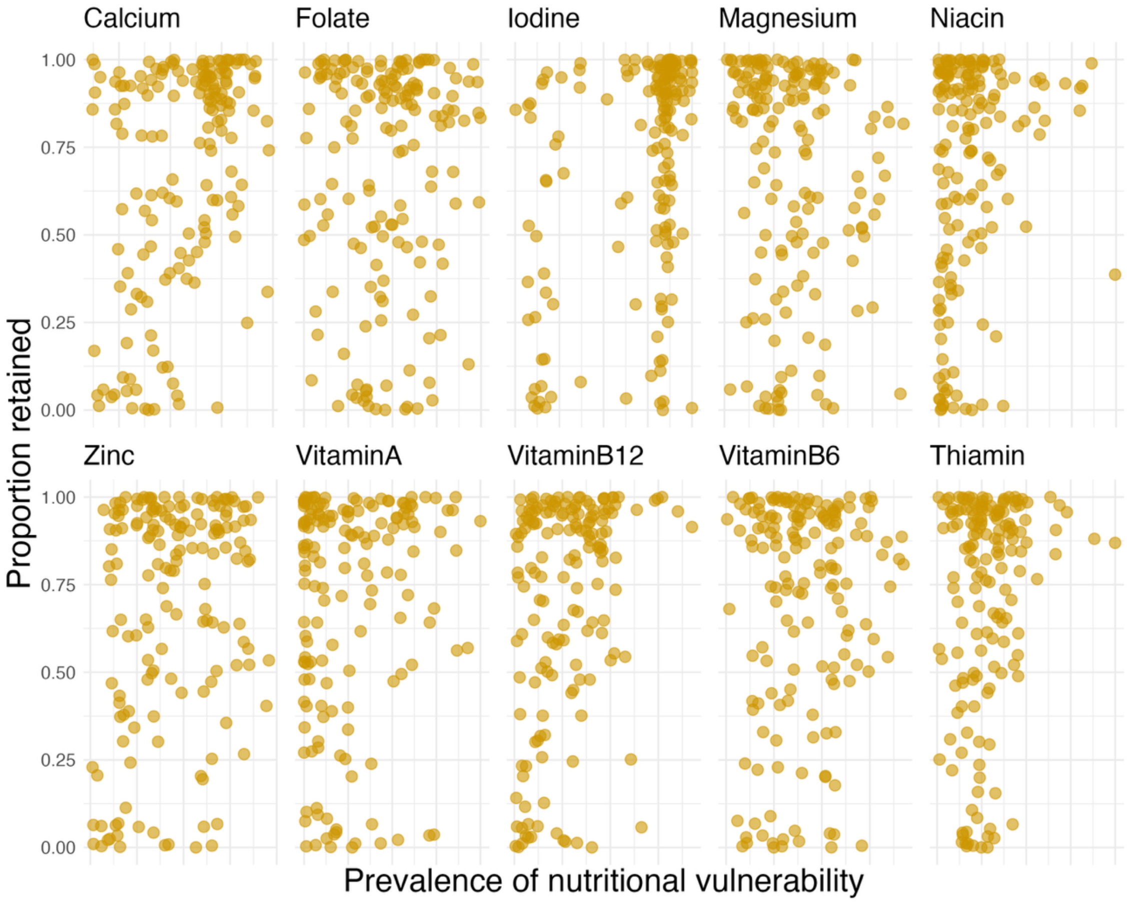

Figure S1. The proportion of countries' domestically retained nutrient supply of aquaculture products by prevalence of nutritional vulnerability. Each point represents a country average.

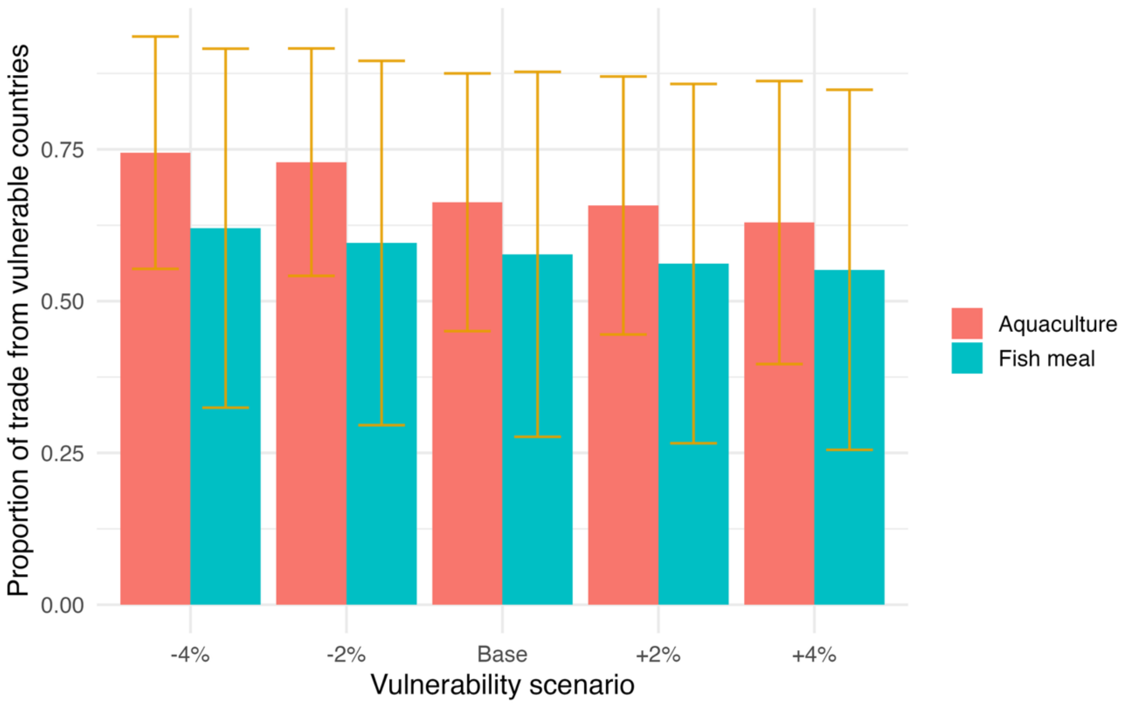

Figure S2. Proportion of traded nutrients from vulnerable countries for fishmeal and aquaculture.

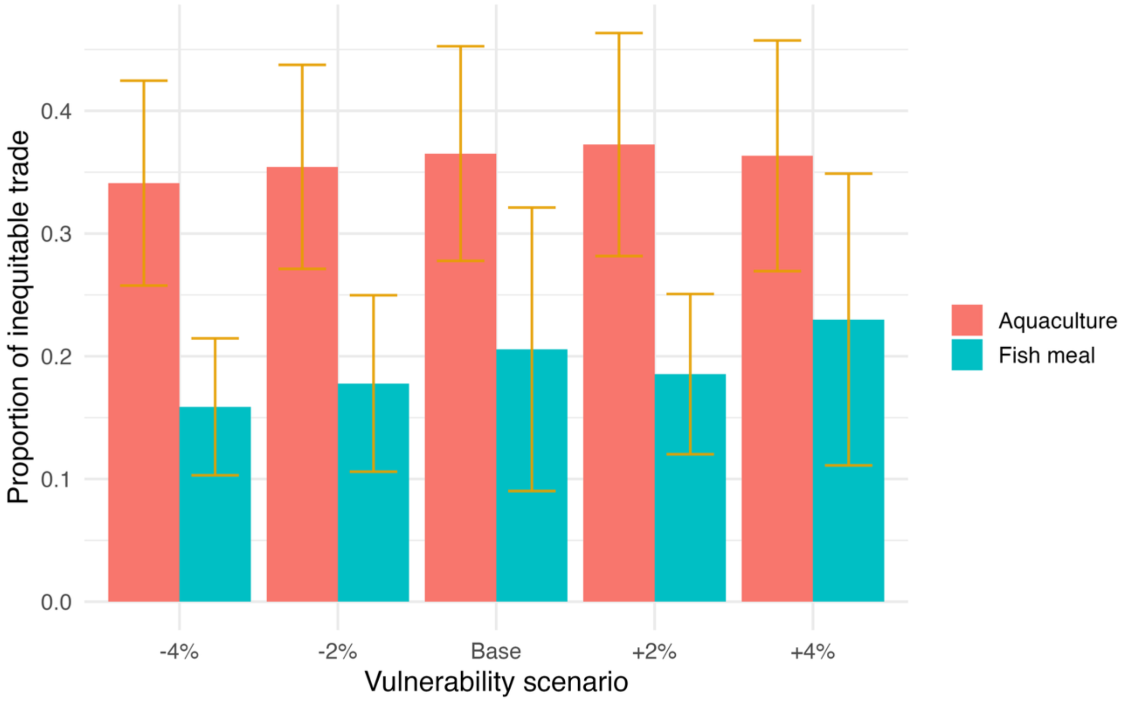

Figure S3. Proportion of traded nutrients from vulnerable countries for fishmeal and aquaculture.

(A) Calcium

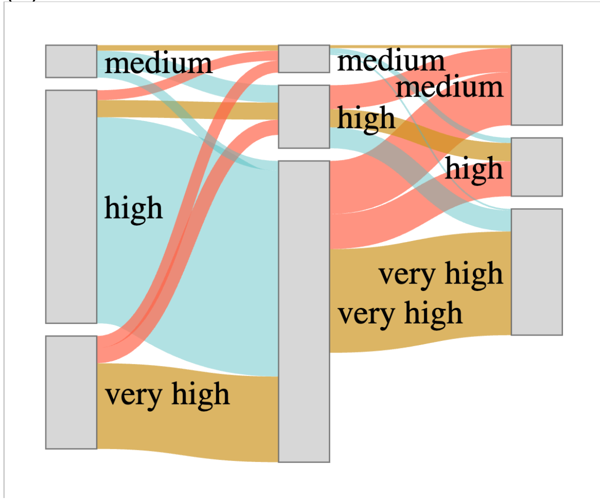

(B) Folate

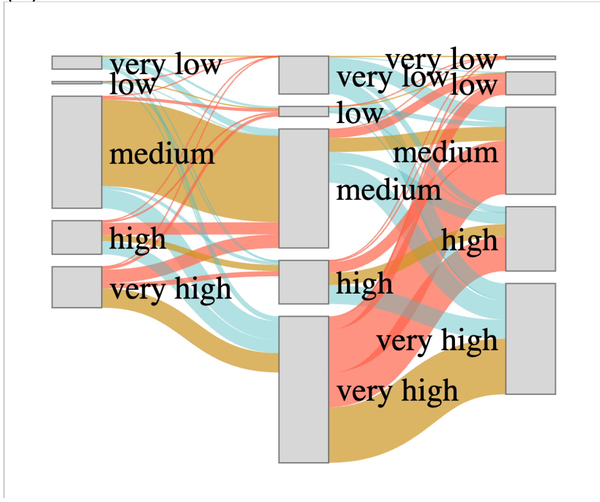

(C) Iodine

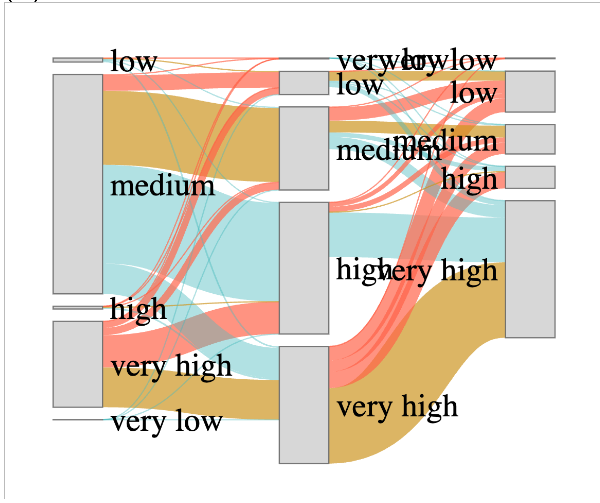

(D) Magnesium

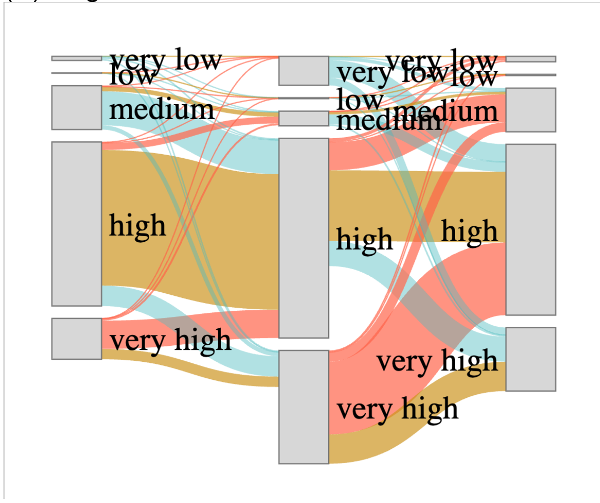

(E) Selenium

(F) Thiamin

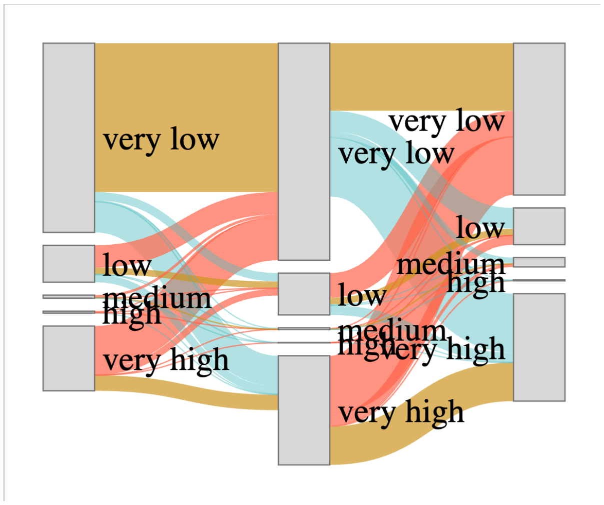

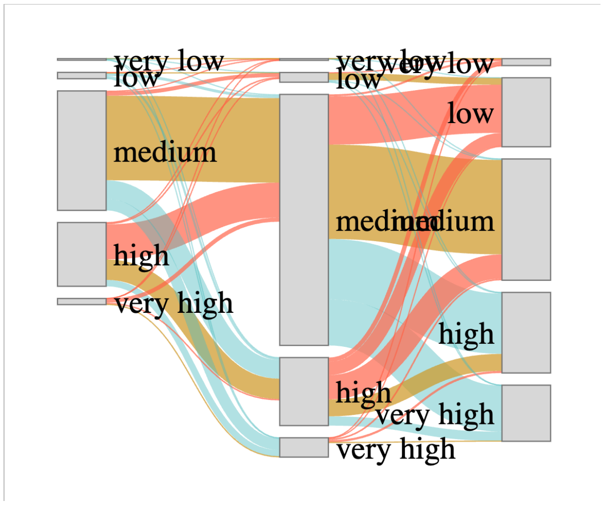

(G) Vitamin A

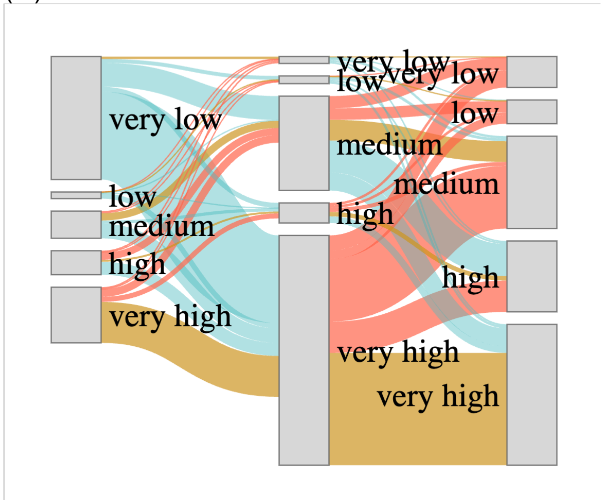

(H) Vitamin B_2

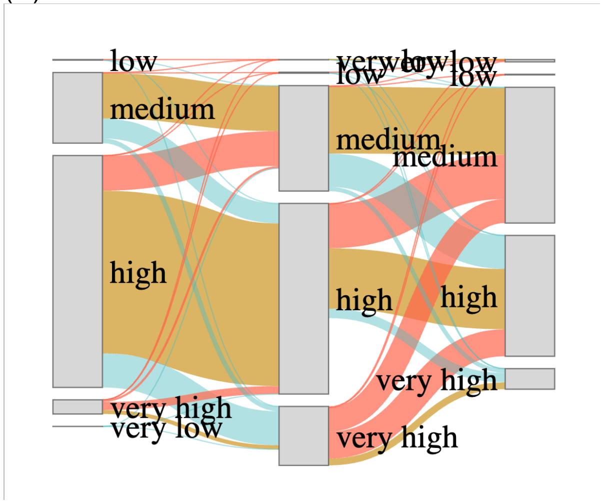

(I) Vitamin B6

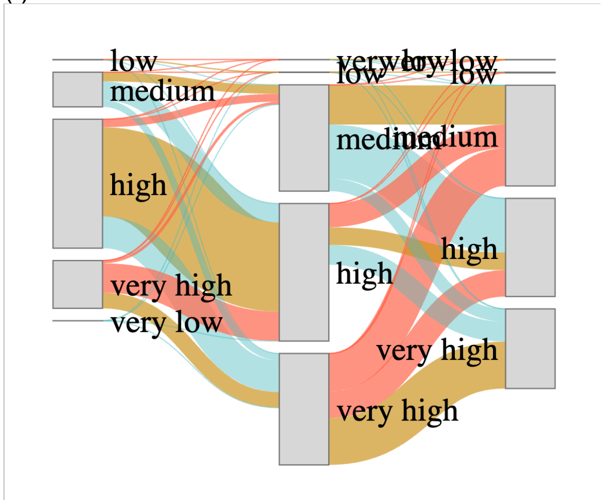

(J) Zinc

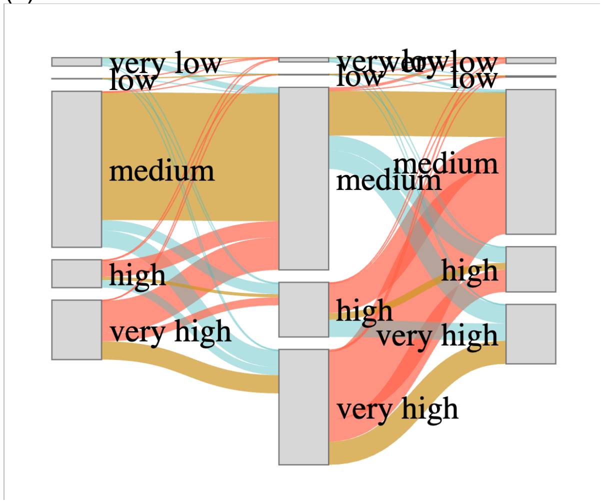

Figure S4. Aquaculture-related trade of remaining nutrients. Sankey diagrams represent nutrient trade flows from countries producing fishmeal (left nodes) to aquaculture-producing (middle nodes) and aquaculture-consuming countries (right nodes). Prevalence of nutritional vulnerability

is measured by nutrient intake inadequacies and referenced by categories: 'very high' countries with >50% of the population with nutrient intake inadequacies; 'high', 25 < % ≤ 50; 'medium',10 < % ≤ 25; 'low', 5 < % ≤ 10; 'very low', ≤5%. Link color indicates trade flows from countries with higher to lower (red), same (yellow), and lower to higher (blue) intake inadequacy. Link width indicates the trade flow quantities by nutrient.

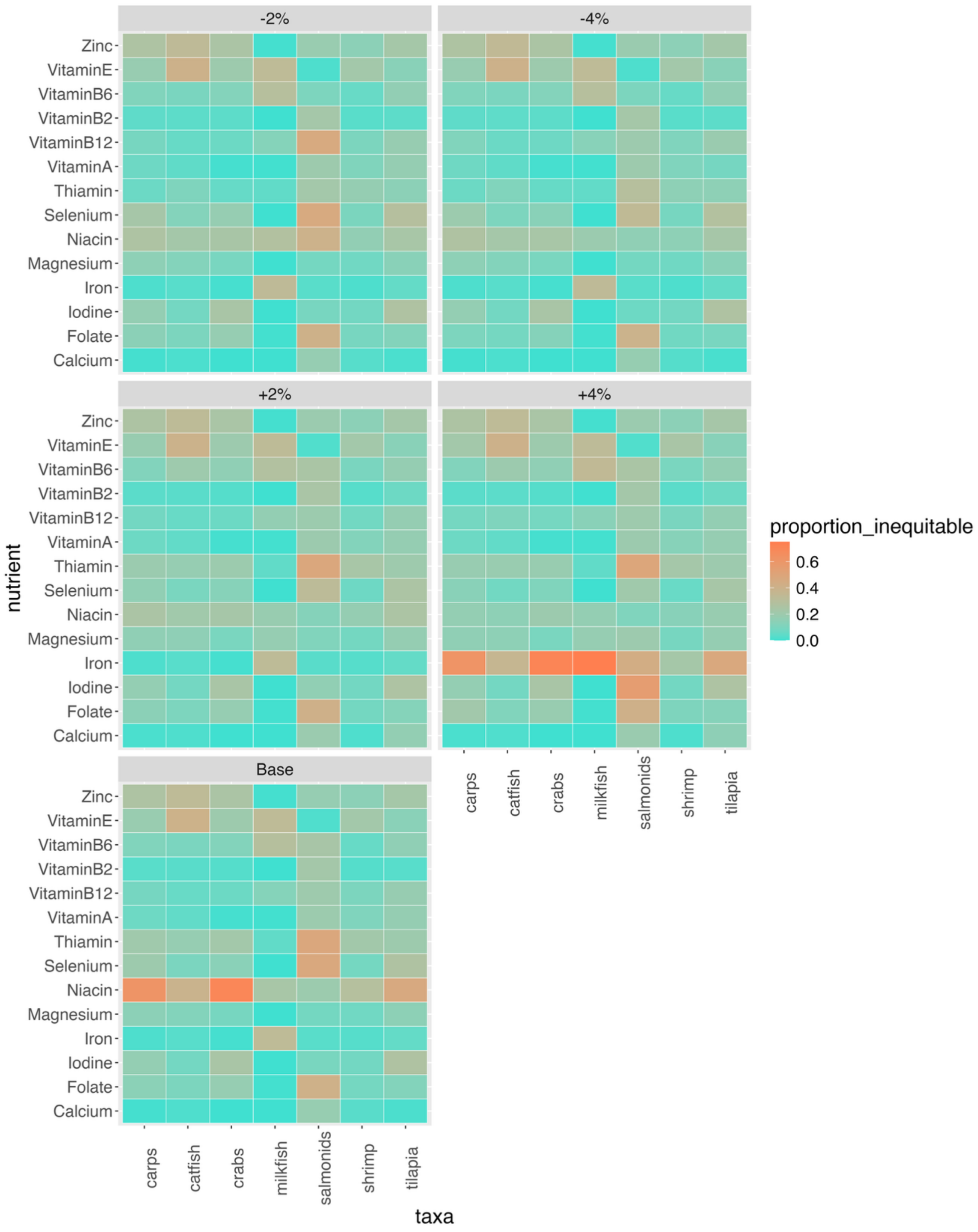

Figure S5. Inequity in fishmeal trade flows. Heatmaps show the proportion of traded volume from more to less vulnerable countries by taxa and nutrient. Fishmeal trade flows consist of wild-caught species used as input for the production of aquaculture taxa specified on the horizontal axis. Each heatmap represents a different country vulnerability thresholds see Table S3. Values have been averaged across trade partners and years.

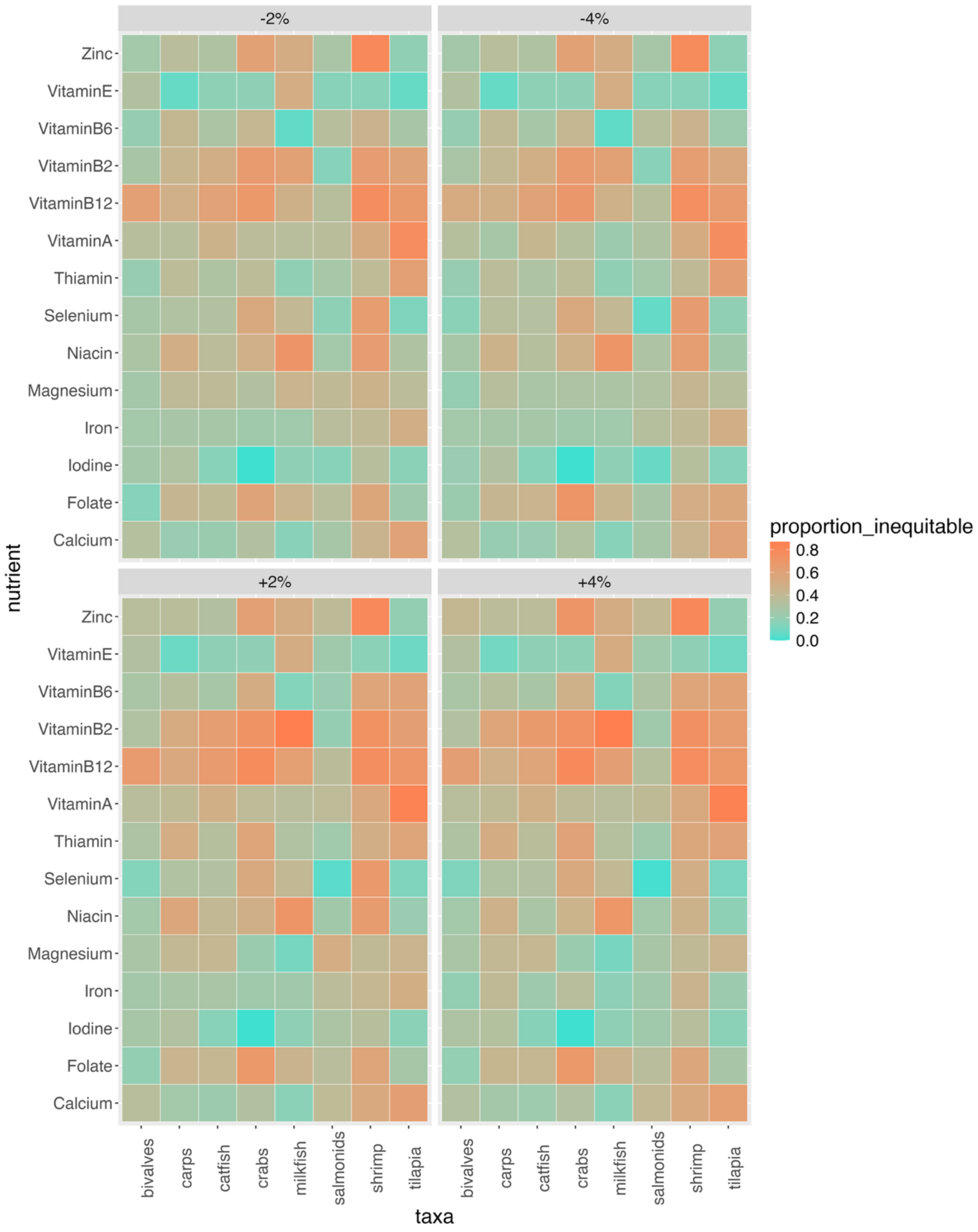

Figure S6. Inequity in aquaculture taxa trade flows. Heatmaps show the proportion of traded volume from more to less vulnerable countries by taxa and nutrient. Each heatmap represents a different country vulnerability thresholds see Table S3. Values have been averaged across trade partners and years.

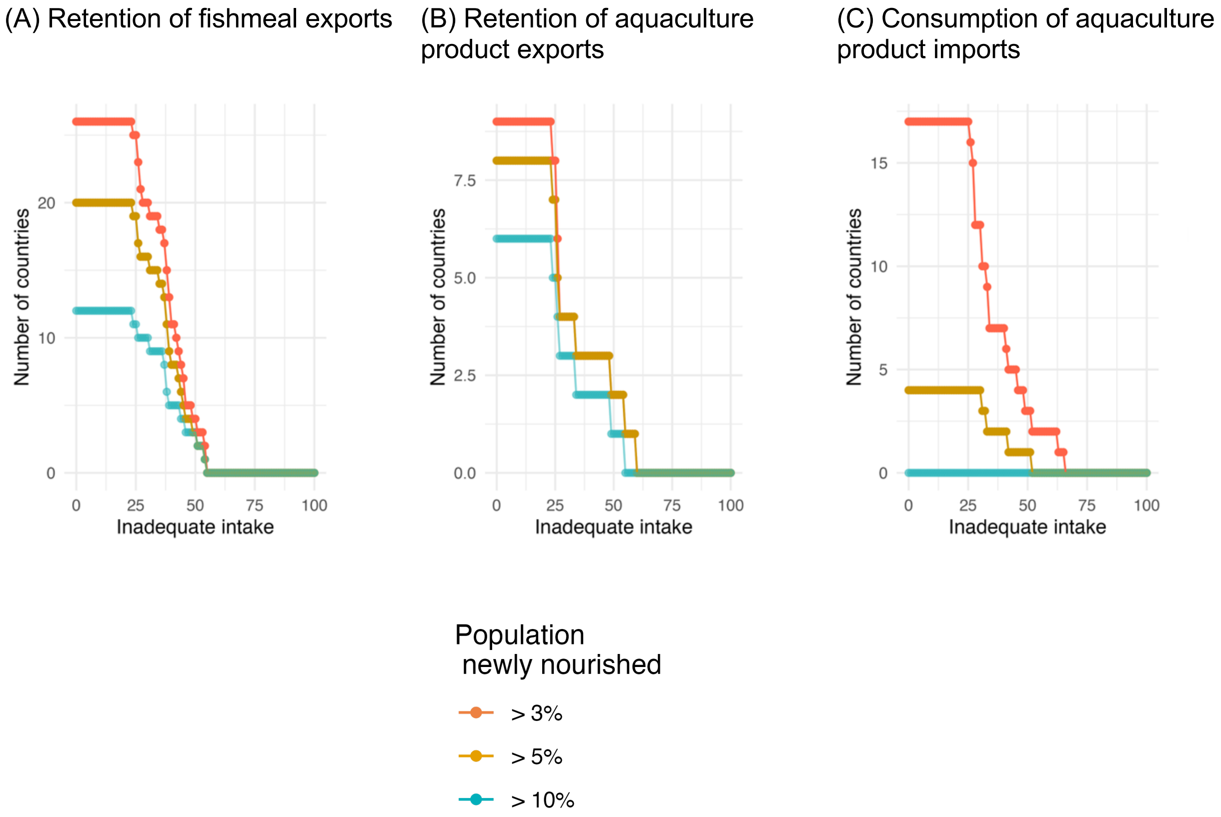

Figure S7. Number of countries that could have newly met individual nutrient needs (i.e., the average number of people with inadequate nutrient intake that could meet annual nutrient needs of 14 nutrients) for 3%, 5%, and 10% of their nutrient-vulnerable population. Left figure shows retaining and directly consuming fishmeal exports (A) and middle figure aquaculture product exports (B). Right figure shows consuming aquaculture product exports (C).

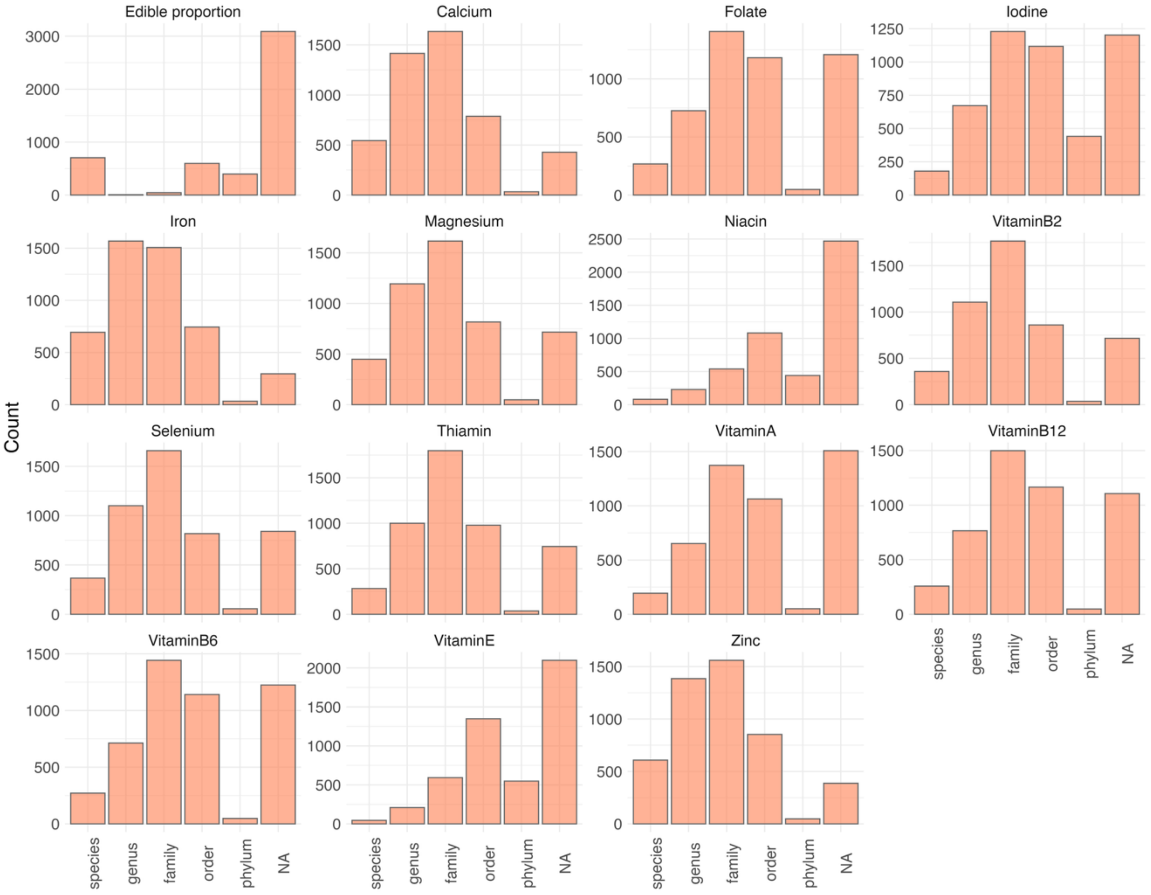

Figure S8. Taxonomic level of matches of edible proportion and between AFCD and ARTIS databases of nutrients.

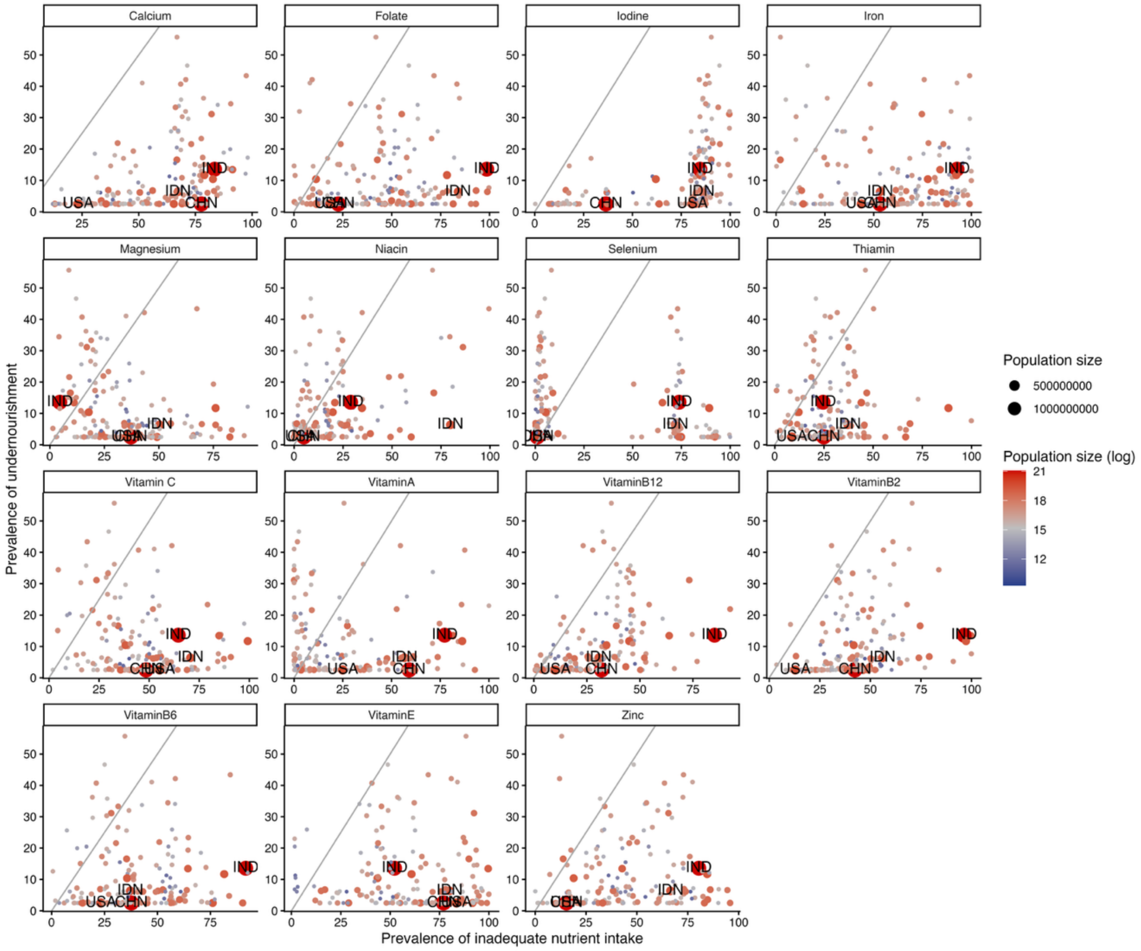

Figure S9. Contrasting prevalence of inadequate intake and undernourishment by country. Prevalence of inadequate nutrient intake from Passarelli et al. 2024 with underlying population data from 2018 and prevalence of undernourishment biannual indicator between 2014 and 2020 from FAO 2023.

Table S1. Sensitivity tests for key statistics.

Result section	Statistic	Baseline value (main text)	Sensitivity test (changed variables)	Values from sensitivity test
2.1	Average annual produced weight from aquaculture	36.1 million tonnes	Live weight values	79 million tonnes
2.1	Average annual nutrient needs produced by global aquaculture	347 million individuals	Live weight values	831 million individuals
			DRI values for pregnant individuals	289 million individuals
			DRI values for lactating individuals	300 million individuals
2.4	Number of individuals potentially meeting nutrient needs from actual farmed fish imports	39.1 million individuals	Live weight values	92.0 million individuals
			DRI values for pregnant individuals	37.5 million individuals
			DRI values for lactating individuals	39.1 million individuals
2.4	Number of individuals potentially meeting nutrient needs from hypothetical retention of farmed fish	31.2 million individuals	Live weight values	73.0 million individuals
			DRI values for pregnant individuals	30.2 million individuals
			DRI values for lactating individuals	31.2 million individuals
2.4	Number of individuals potentially meeting nutrient needs from hypothetical retention and direct consumption of aquatic species used in fishmeal	31.0 million individuals	Live weight values	47.0 million individuals
			Reducing fishmeal by 34% from trimmings	23.3 million
			DRI values for pregnant individuals	27.6 million individuals
			DRI values for lactating individuals	28.3 million individuals

Table S2. Nutrients and total weight produced by aquaculture and fisheries.

Nutrients in annual RDAs	Aquaculture	Fisheries	Proportion of total from aquaculture
Calcium	52,856,296	68,389,614	0.44
Folate	38,428,867	35,651,688	0.52
Iodine	238,486,652	398,908,884	0.37
Iron	100,857,155	150,186,493	0.40
Magnesium	120,799,219	178,550,959	0.40
Niacin	521,481,116	727,280,829	0.42
Vitamin B_2	114,275,667	210,815,655	0.35
Selenium	467,357,559	1,147,557,461	0.29
Thiamin	129,185,420	117,051,943	0.52
Vitamin A	17,143,509	29,569,251	0.37
Vitamin B_{12}	2,668,940,304	3,301,921,939	0.45
Vitamin B_6	156,799,061	369,269,457	0.30
Vitamin E	47,700,546	48,123,312	0.50
Zinc	184,423,790	240,796,449	0.43
**Weight in tonnes**			
Live weight	78,983,241	93,739,667	0.46
Edible weight	36,123,472	48,930,759	0.42

Table S3. Average country nutrient vulnerability (measured in percent population with inadequate intake) for countries with gains and losses per nutrient.

	Average vulnerability for countries with nutrient losses	Average vulnerability for countries with nutrient gains
**Calcium**	56.88	42.98
**Folate**	44.91	45.03
**Iodine**	49.59	61.20
**Iron**	71.53	60.34
**Magnesium**	43.13	41.74
**Niacin**	15.29	18.55
**Selenium**	23.56	27.62
**Thiamin**	27.41	28.65
**Vitamin A**	35.45	34.57
**Vitamin B_{12}**	32.31	11.99
**Vitamin B_2**	41.03	27.73
**Vitamin B_6**	43.82	42.32
**Vitamin E**	66.75	68.37
**Zinc**	38.92	32.91

Table S4. Country ranks in nutrient gains and losses measured in individual nutrient intake needs. Note, in the 34% trimmings reduction scenario Norway and Peru switch ranks.

Rank #	Country	Median gains and losses	Median gains and losses using live weights	Country incl. 34% trimmings reductions	Median gains and losses incl. 34% trimmings reductions
1	USA	2,893,600	5,749,596	USA	2,335,613
2	Japan	1,685,418	3,411,309	Japan	1,333,959
3	France	738,031	1,548,473	France	599,378
190	Norway	-3,220,748	5,559,505	Chile	-2,903,730
191	Chile	-3,230,962	6,252,599	Norway	-3,146,279
192	Peru	-5,686,381	10,386,920	Peru	-3,763,560

Table S5. Alternative estimates of trade equity using absolute values and a threshold of 5% higher and lower for the category 'trade with same vulnerability' (Methods 4.4).

Type	Nutrient	Proportion equitable trade	Proportion inequitable trade	Proportion trade with same vulnerability
**Aquaculture**	Calcium	0.22	0.66	0.12
	Folate	0.35	0.53	0.12
	Iodine	0.29	0.35	0.36
	Iron	0.38	0.52	0.10
	Magnesium	0.39	0.46	0.15
	Niacin	0.36	0.37	0.27
	Vitamin B_2	0.36	0.46	0.19
	Selenium	0.29	0.29	0.42
	Thiamin	0.34	0.43	0.22
	Vitamin A	0.29	0.53	0.18
	Vitamin B_{12}	0.26	0.56	0.18
	Vitamin B_6	0.43	0.45	0.13
	Vitamin E	0.48	0.35	0.17
	Zinc	0.31	0.52	0.17
	**Average**	**0.34**	**0.46**	**0.20**
**Fishmeal**	Calcium	0.69	0.19	0.12
	Folate	0.61	0.32	0.06
	Iodine	0.36	0.22	0.42

	Iron	0.19	0.67	0.14
	Magnesium	0.26	0.19	0.55
	Niacin	0.25	0.20	0.56
	Vitamin B_2	0.30	0.19	0.51
	Selenium	0.14	0.24	0.62
	Thiamin	0.36	0.26	0.38
	Vitamin A	0.63	0.28	0.10
	Vitamin B_{12}	0.56	0.29	0.15
	Vitamin B_6	0.32	0.25	0.43
	Vitamin E	0.24	0.65	0.11
	Zinc	0.25	0.25	0.50
	**Average**	**0.37**	**0.30**	**0.33**

Table S6. Databases and variables used in analysis.

Database	Source / Version	Variable and use description
Aquatic Food Composition Database (AFCD)	C. D. Golden, et al., "Aquatic Food Composition Database". AFCD. Harvard Dataverse. https://doi.org/10.7910/DVN/KI0NYM. Accessed: 07/08/2025	218,975 observations of 12 variables. Used variables include taxonomic levels, food part, processing, and nutrient concentrations
Aquatic Resource Trade in Species database (ARTIS)	J. Gephart, R. Agrawal Bejarano, "Globalization of wild capture and farmed aquatic foods". ARTIS, https://doi.org/10.5281/zenodo.10034319. Accessed: 12/01/2024	Fishmeal: 962,521 observations of 14 variables. Used variables include source and destination country alpha iso codes, scientific name, method, live weight, and year<br>Aquaculture: 625,767 observations of 14 variables. Used variables include consumer and source iso, scientific name, method, year, and live weight
FAO Fisheries and Aquaculture Production data	FAO, "Fishery and Aquaculture Statistics. Global production by production source 1950-2021 (FishStatJ)." FAO Fisheries and Aquaculture Division, 2023. Accessed: 10/01/2023	58,864 observations of 6 variables. Used variables include country, iso-alpha 3 country code, method, habitat, year, and live weight. Used to calculate the proportion of aquatic foods produced in aquaculture
Inadequate intake data	S. Passarelli, *et al.*, Global estimation of dietary micronutrient inadequacies: a modelling analysis. *The Lancet Global Health* 12, e1590–e1599 (2024).	118,592 observations of 15 variables. Used variables include iso alpha-3 code, nutrient, sex, age range, summary exposure value, number of deficient people
Blue Food Assessment Feed Conversion Ratios	J. A. Gephart, *et al.*, Environmental performance of blue foods. *Nature* 597, 360–365 (2021).	12 median values (of 372 observations) of 6 variables. Used variables include taxa, FCR, and fishmeal and oil in feed
Aquamodelr Feed Conversion Ratio and On-feed Proportion data	T. Cashion, "Aquamodelr package. Modelling Aquaculture Feed Use Globally". GitHub. https://github.com/timcashion/aquamodelr/tree/master. Accessed: 12/01/2023	14,208 observations of 6 variables. Used variables include country, taxon, type variable (FCR, fishmeal and oil in feed, and percent on feed variables), measure, and value. We only used data on crustaceans

Table S7. Nutrient intake classifications.

Nutrient intake classifications	Cut-off values in percent of total population
Very high inadequate intake	>50%
High inadequate intake	>25 to ≤ 50
Medium inadequate intake	>10 to ≤ 25
Low inadequate intake	> 5 to ≤ 10
Very low inadequate intake	≤5%

Table S8. Country vulnerability thresholds binned by cut-off values.

Scenarios of cut-off values	Cut-off values: very low-low-medium-high-very high
Base scenario (adopted from Nash et al. 2022)	0-5-10-25-50-100
-4%	0-1-6-21-46-100
-2%	0-3-8-23-48-100
+2%	0-7-12-27-52-100
+4%	0-9-14-29-54-100
